# In Silico Target Prediction of Overexpressed microRNAs from LPS-Challenged Zebrafish (*Danio rerio*) Treated with the Novel Anti-Inflammatory Peptide *Tn*P

**DOI:** 10.3390/ijms22137117

**Published:** 2021-07-01

**Authors:** Geonildo R. Disner, Maria A. P. Falcão, Carla Lima, Monica Lopes-Ferreira

**Affiliations:** Immunoregulation Unit of the Laboratory of Applied Toxinology (CeTICS/FAPESP), Butantan Institute, São Paulo 05503-900, Brazil; geonildo.disner@butantan.gov.br (G.R.D.); maria.falcao@butantan.gov.br (M.A.P.F.); carla.lima@butantan.gov.br (C.L.)

**Keywords:** *Tn*P, inflammation, preclinical toxicological studies, epigenetic, miRNAs, bioinformatics, ontology

## Abstract

miRNAs regulate gene expression post-transcriptionally in various processes, e.g., immunity, development, and diseases. Since their experimental analysis is complex, in silico target prediction is important for directing investigations. *Tn*P is a candidate peptide for anti-inflammatory therapy, first discovered in the venom of *Thalassophryne nattereri*, which led to miRNAs overexpression in LPS-inflamed zebrafish post-treatment. This work aimed to predict miR-21, miR-122, miR-731, and miR-26 targets using overlapped results of DIANA microT-CDS and TargetScanFish software. This study described 513 miRNAs targets using highly specific thresholds. Using Gene Ontology over-representation analysis, we identified their main roles in regulating gene expression, neurogenesis, DNA-binding, transcription regulation, immune system process, and inflammatory response. miRNAs act in post-transcriptional regulation, but we revealed that their targets are strongly related to expression regulation at the transcriptional level, e.g., transcription factors proteins. A few predicted genes participated concomitantly in many biological processes and molecular functions, such as *foxo3a*, *rbpjb*, *rxrbb*, *tyrobp*, *hes6*, *zic5*, *smad1*, *e2f7*, and *npas4a*. Others were particularly involved in innate immunity regulation: *il17a/f2*, *pik3r3b*, and *nlrc6*. Together, these findings not only provide new insights into the miRNAs mode of action but also raise hope for *Tn*P therapy and may direct future experimental investigations.

## 1. Introduction

Epigenetic mechanisms control numerous functions throughout the body, from cell fate determination in development to immune responses and inflammation, including direct actions in macrophages, T and B cells, and microglia [1]. Epigenetic modifications can be mediated by three main mechanisms: DNA methylation, histone modifications, and non-coding RNA (ncRNA). These adjustments influence chromatin structure, altering gene transcription and translation.

The microRNA (miRNA) discovery has added a new complexity level to understanding homeostatic processes and disease control [2]. The miRNAs are 18–25 nucleotides (nt) long molecules, single-stranded, that generally bind to the 3′ untranslated regions (UTRs) of target messenger RNAs (mRNAs), particularly with their *seed* region consisting of a short stretch (2–8 nt) in the sequence 5′ end. In addition to their role in gene silencing and translational repression by binding to target mRNAs, miRNAs also mediate transcript degradation and nucleic acid-binding proteins [3].

Each miRNA can bind to several types of mRNAs, and the same mRNA can be targeted by different miRNAs in a concerted manner [4]. miRNAs act from the life cycle in eukaryotic cells to biological processes, such as molecular and metabolic processes, immunity, stress response, cell proliferation, differentiation and maturation, and response to environmental stimulus and diseases, such as cancer and neuroinflammation, including neurodegenerative and autoimmune diseases [5,6]. The large number of miRNAs and their broad target ability suggest a complex regulatory network to fine-tune the gene expression [7,8]. Therefore, identifying those target genes is essential to understanding the complex regulatory pathways.

As miRNAs are highly conserved evolutionarily in vertebrates, studies using different model organisms can bring valuable information about their functions in human disease progress. The teleost zebrafish (*Danio rerio*) is a versatile model organism for investigating cellular and molecular mechanisms adjacent to several pathological processes and for monitoring environmental contaminants [9,10,11]. Moreover, zebrafish have been used to evaluate candidate therapeutic agents through preclinical toxicological studies required by the pharmaceutical industry in the initial stage of drug discovery [12,13,14,15].

In the last decade, zebrafish has particularly been applied to the study of miRNAs functions; so far, 355 precursor sequences and 373 mature miRNAs are described in zebrafish [16,17,18]. Growing evidence shows that miRNAs play important roles in regulating inflammation in zebrafish [19,20,21,22]. Furthermore, in zebrafish, the lipopolysaccharide (LPS) challenge provides a robust and safe way to test anti-inflammatory drugs as well as their pathways under controlled conditions and to address basic scientific questions. Bacterial LPS is a potent endotoxin that stimulates innate immunity, inducing the acute phase response, systemic inflammation, and the production of proinflammatory cytokines.

The *Tn*P family invention, currently patented in several countries, refers to synthetic peptides uncovered from the venom of the Brazilian venomous toadfish *Thalassophryne nattereri* with anti-inflammatory and anti-allergic activities, containing a sequence of 13 L-amino acids in their primary structure (C_63_H_114_N_22_O_13_S_4_, H–I-P-R-C-R-K-M-P-G-V-K-M-C–NH2, with a disulfide bond between Cys4 and Cys13; 1514.8 Da; pI 10.63). *Tn*P has remarkable resistance to the action of proteolytic enzymes such as trypsin and pepsin, and its high solubility allows it to be quickly absorbed. In mice, we observed that immunization with *Tn*P at different doses, in the presence or absence of adjuvant, does not generate specific antibodies and is therefore not immunogenic [23]. *Tn*P is in a preclinical development stage, indicated for chronic inflammatory diseases such as asthma and multiple sclerosis (MS).

Our recent findings show the valuable potential of synthetic peptides of the patented *Tn*P family for controlling neuroinflammation and preventing demyelination in diseases such as multiple sclerosis due to its systemic ability to interfere in the dynamic circuit of immune cell groups as well as locally in the central nervous system (CNS). *Tn*P amends experimental autoimmune encephalomyelitis (EAE) in an IL-10-dependent way, controls leukocyte infiltration, and inhibits demyelination, reducing the disease severity and delaying symptoms. In addition, microglia expansion and the activity of matrix metalloproteinase-9 (MMP-9) by F4/80^+^ macrophages were decreased. *Tn*P modulates the encephalitogenic CD4^+^ T cells, reducing in the CNS-infiltrating IFN-γ-producing Th1 and IL-17A-producing Th17 cells. *Tn*P also blocks the proinflammatory cytokines production in the spleen and promotes the emergence of functional regulatory T cells (Treg) in the spleen and CNS [24].

With a view to advancing the preclinical development chain, investigations using RNA-sequencing-based transcriptome assay revealed that *Tn*P treatment in zebrafish larvae stimulated by LPS altered the expression levels of several genes; among the upregulated *Danio rerio* mature miRNAs (dre-miR) were dre-miR-21, dre-miR-122, dre-miR-731, and dre-miR-26 [25], herein just referred to as miR-21, miR-122, miR-731, and miR-26. These findings led us to hypothesize that the reduction in the translation of mRNAs that encode inflammatory response-related genes promoted by miRNAs could be one of the molecular bases of the *Tn*P anti-inflammatory effect.

To expand the knowledge about the *Tn*P immunomodulatory mechanisms, we used in silico approaches to analyze the features of miR-21, miR-122, miR-731, and miR-26 as likely targeting multiple genes. For this, DIANA microT-CDS (v. 5.0, http://diana.imis.athena-innovation.gr/DianaTools/index.php?r=microT_CDS/index, accessed on 29 April 2021, Thessaly, Greece) and TargetScanFish (v. 6.2, http://www.targetscan.org/fish_62/, accessed on 29 April 2021, Cambridge, MA, USA) tools were used to provide insights into what genes might be regulated by the TnP-mediated overexpressed miRNAs. Ultimately, besides being controlled by drugs, the miRNAs’ nature makes them powerful candidates as therapeutics, either through miRNA mimics or as targets of therapeutics by antimiRs.

## 2. Results

### 2.1. miRNAs Putative Target Genes

Owing to the biological significance of miRNAs, many bioinformatics algorithms have been developed for data warehousing (miRBase, miRTarBase) and miRNA target predictions (DIANA-TarBase, TargetScan) [26,27,28]. However, the in silico target prediction tools remain with a substantial false-positive rate because they mainly use sequence complementarity and assume structural stability to predict specific targets of miRNA [29], which might be bypassed by establishing thresholds with high specificity and comparing predictions among software. Moreover, the most significant contribution in terms of complementarity is related predominantly to the seed portion. In addition, experimental validation of the miRNA targets necessary to strengthen the prediction reproducibility and credibility must be performed.

Here, to elucidate the functions of the miRNAs correlated with the *Tn*P anti-inflammatory effect, two software packages, DIANA microT-CDS 5.0 and TargetScanFish 6.2, were programmed to predict the putative miRNA:mRNA interactions. The target gene localizations were widely overspread along the 25 zebrafish chromosomes. The ones housing more targets from the list were the 6 and the 16, with 34 (6.63%) and 32 (6.24%) genes, respectively. On the other hand, chromosome 25, the smallest one, contained only 9 targets (1.75%). On average, each zebrafish chromosome carried 20.5 predicted genes, according to the analysis. From the resulting data, most of the genes had annotation, which helped to assign functional characteristics; just about 4% had scarce or unavailable information.

**Figure 1:**
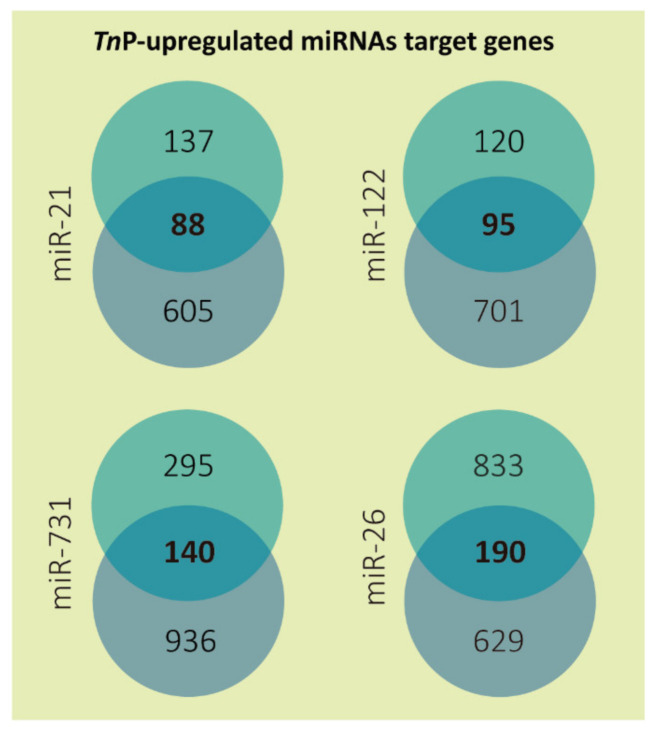
Number of predicted target genes for each TnP-induced microRNA. The targets predicted by DIANA microT-CDS (miTG score > 0.7) are presented in the top circles and by TargetScanFish (score + context < −0.2) in the bottom circles; in the center is shown the overlapped results representing only the genes mutually found. The miR-26 targets from DIANA microT-CDS are the sum of miR-26a and 26b predictions, excluding duplicates.

Altogether, 513 putative targets of the four selected miRNAs were achieved after applying the low sensitivity and high specificity selection criteria (Figure 1; Supplementary Table S1). Overlaps between both tools resulted in a total of 88 predicted genes to miR-21, 95 to miR-122, 140 to miR-731, and 190 to miR-26. The miR-26 may have presented more target genes because this miRNA family has more than one member. In zebrafish, the highly conserved miR-26 family consists of miR-26a-1, miR-26a-2, and miR-26b. The mature miR-26a-1 and miR-26a-2 miRNA have the same sequence, which differs from miR-26b by only one nucleotide [30].

**Figure 2:**
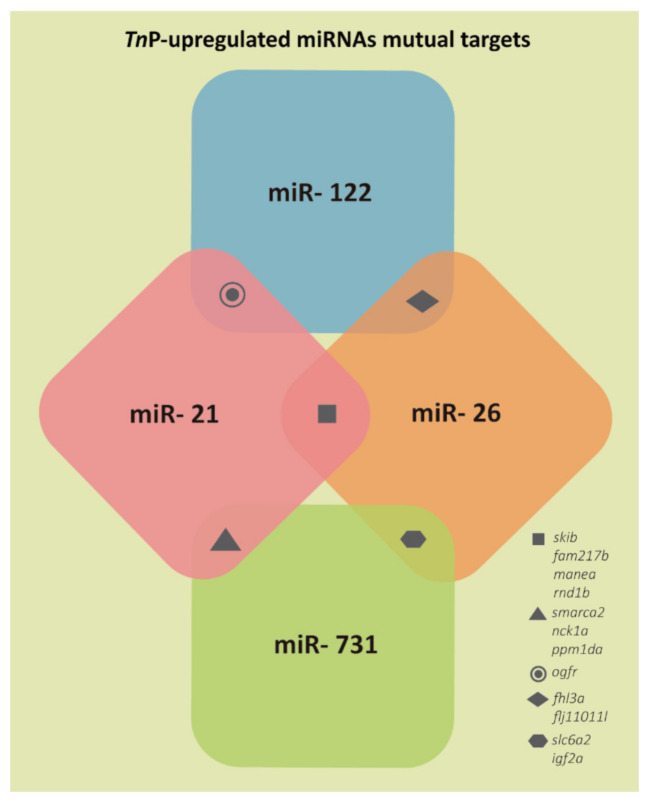
Representation of the few target genes mutually predicted for two of the *Tn*P-induced miRNAs investigated.

The results presented in Figure 2 demonstrate that only 12 genes could be targeted by more than one of the miRNAs assessed. Namely, *orfr* could be targeted by miR-21 and miR-122; *fhl3a* and *flj11011l* by miR-122 and miR-26; *slc6a2* and *igf2a* by miR-26 and miR-731; *smarca2*, *nck1a*, and *ppm1da* by miR-731 and miR-21; and *skib*, *fam217b*, *manea*, and *rnd1b* by miR-21 and miR-26.

### 2.2. Biological Significance of Identified Putative Target Genes

**Figure 3:**
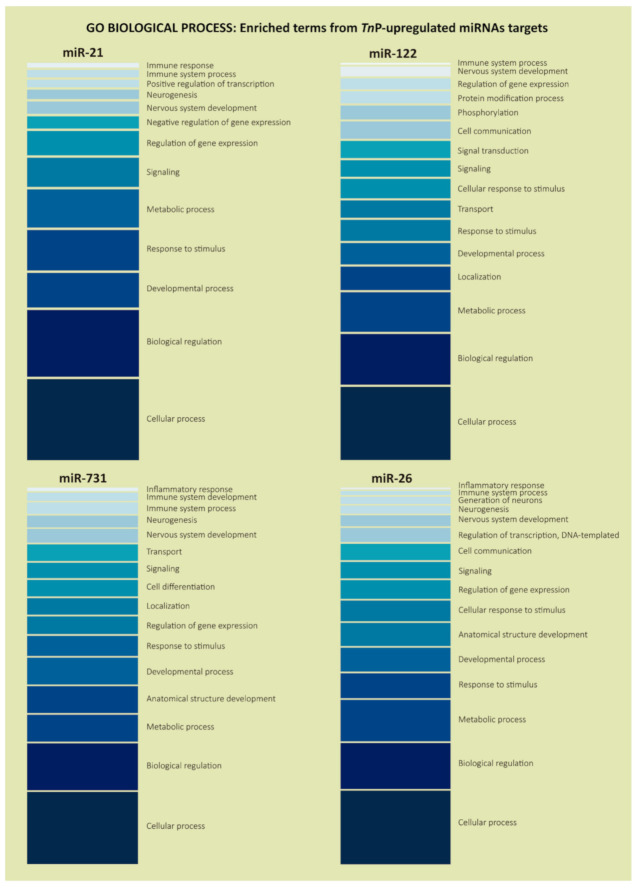
Gene Ontology enrichment analysis in the “Biological Process” category for the predicted target genes of *Tn*P-induced miR-21, miR-122, miR-731, and miR-26. It illustrates the most enriched terms and some classes relevant for the experimental condition. The color scale, from darker to lighter, represents the terms with more genes in it (**bottom**) or with just a few genes (**top**), based on the enrichment analysis.

We used the PANTHER system for explaining the biological process, molecular function, and cellular components, where the gene ontology (GO) term enrichment assay selected a non-redundant set of annotations with maximal mutual information to miR-21, miR-122, miR-731, and miR-26 target set inputs. The PANTHER Classification System is designed to be a comprehensive platform for analyzing gene function on a genome-wide scale; since the initial release in 2003, it has become one of the most popular online resources for genome-wide data analysis. Thus, this analysis allowed us to find over-represented biological processes categories as cellular process (GO:0009987), biological regulation (GO:0065007), metabolic process (GO:0008152), developmental process (GO:0032502), response to stimulus (GO:0050896), signaling (GO:0023052), and regulation of gene expression (GO:0010468), in descending order (Figure 3).

According to the role of *Tn*P in neuroinflammation, nervous system development (GO:0007399) and neurogenesis (GO:0022008) ontology terms were selected. Functions related to these couple of terms were highly represented with plenty of genes from the prediction list of all four miRNAs (58 in total), notably the miR-731, that presented more target genes (around 40%). Overall, those genes with predicted functional engagement in the neuronal process were: *gsna*, *tnn*, *rbpjb*, *nrsn1l*, *hes6*, *myrf*, *tcf7l1b*, and *egr1* (miR-21 targets); *dvl2*, *omgb*, *zc4h2*, *col6a2*, *dpf3*, *ralgapa1*, *acsl3b*, and *lingo2a* (miR-122 targets); *sec63*, *draxin*, *ret*, *gpm6bb*, *sebox*, *ephb2b*, *znf423*, *efna2b*, *rgma*, *hmx3a*, *vsx1*, *neurod2*, *sipa1l1*, *mosmob*, *rab3aa*, *enc1*, *wnt7aa*, *rhogd*, *lrrc15*, *prdm8b*, *grna*, dla, and *bmi1a* (miR-731 targets); *get3*, *loxl3b*, *foxj1b*, *sez6l2*, *rp1l1b*, *tiam1a*, *zic5*, *bves*, *tor1*, *med27*, *sox3*, *gsk3ba*, *npas4a*, *chrna1*, *fzd3a*, slc2a2, *e2f7*, *loxa*, and *col19a1* (miR-26 targets) (Supplementary Table S1).

Then, we focused deeply on immunity parameters as immune system process (GO:0002376), immune response (GO:0006955), immune system development (GO:0002520), and innate immune response (GO:0045087) and their miRNAs target genes, based on Forn-Cuní et al. [31], which analyzed the zebrafish genomic responses after an acute endotoxin stress event.

Although there were just a few genes in each of these GO terms, they emphasize the importance of the miRNAs in immune regulation, which has been the core of our investigations over the last decade, mainly using the zebrafish model. The genes from the prediction list related to immunity were: *mbd3b*, *mhc1zfa*, *tyrobp*, si:*dkey-22i16.9*, and *foxn1* (miR-21); *fgf1a* and *cicb* (miR-122); *sumo1*, *march2*, *angptl2b*, *efna2b*, *ifit15*, *ZBTB11*, *si:dkey-42l23.7*, *relb*, *klf3*, and *bmi1a* (miR-731); *smad1*, *epc2*, *rag2*, *pqbp1*, *tnk2a*, *g3bp1*, and *rnasel3* (miR-26) (Supplementary Table S1).

The GO term inflammatory response (GO:0006954) appeared quietly in the enrichment analysis of miR-26 and miR-731 targets. This function was fundamental once we investigated *Tn*P, a novel peptide, as an anti-inflammatory therapeutic agent. The genes related to this function were *loxl3b*, *ptgs2b* (miR-26), *si:dkey-42l23*.7, and *relb* (miR-731).

**Figure 4:**
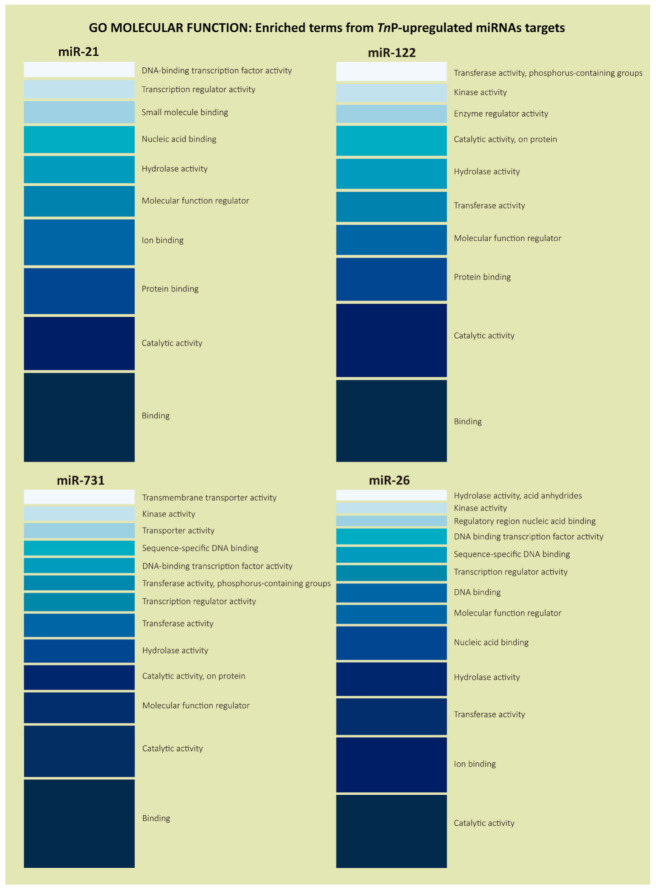
Gene Ontology enrichment analysis in the “Molecular Function” category for the predicted target genes of *Tn*P-induced miR-21, miR-122, miR-731, and miR-26. It illustrates the most enriched terms and some classes relevant for the experimental condition. The color scale, from darker to lighter, represents the terms with more genes in it (**bottom**) or with just a few genes (**top**), based on the enrichment analysis.

Regarding GO molecular function terms, which represent activities that occur at the molecular level, the analysis indicated that many genes were involved in binding (GO:0005488), ion binding (GO:0043167), catalytic activity (GO:0003824), and hydrolase activity (GO:0016787), in addition to the well-represented term transcription regulator activity (GO:0140110), mainly for miR-21, miR-731, and miR-26 (Figure 4).

**Figure 5:**
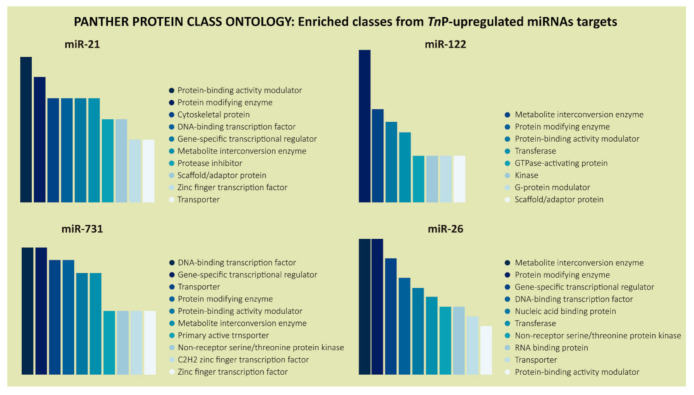
PANTHER classification system in the “Protein Class” category for the predicted target genes of *Tn*P-induced miR-21, miR-122, miR-731, and miR-26. The color scale, from darker to lighter, represents the most common protein classes coded by the putative targets.

Not surprisingly, DNA-binding transcription factor (PC00218) and gene-specific transcriptional regulator (PC00264) came out among the highest-rated protein classes with a fair number of genes in the PANTHER protein class analysis, except for miR-122, which presented a slightly divergent and specific pattern (Figure 5).

**Figure 6:**
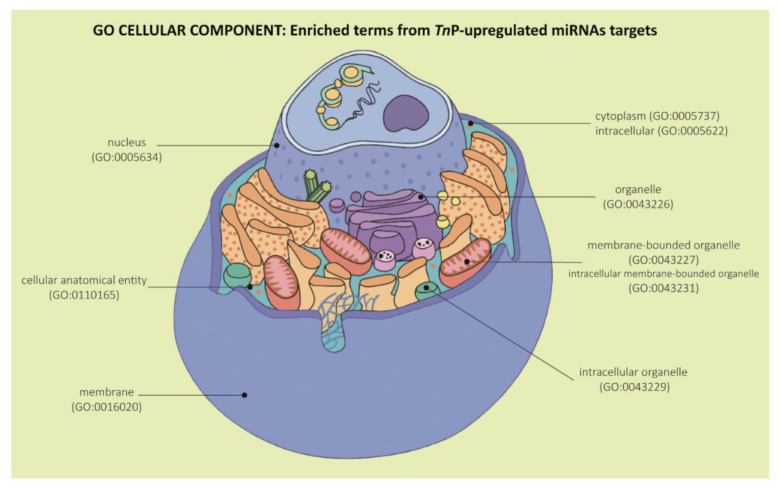
Representation of Gene Ontology enrichment analysis in the “Cellular Component” category for the predicted target genes of *Tn*P-induced miR-21, miR-122, miR-731, and miR-26 altogether. Figure adapted from Cell Anatomy Viewer v. 2.1, Arizona State University.

Ultimately, the GO provides cellular component enriched terms to describe the locations relative to cellular anatomical structures in which the predicted gene products perform their roles. Our over-represented cellular components analyses for all the *Tn*P-upregulated miRNAs were mainly linked to cellular anatomical entity (GO:0110165), intracellular (GO:0005622), organelle (GO:0043226), intracellular organelle (GO:0043229), membrane-bounded organelle (GO:0043227), intracellular membrane-bounded organelle (GO:0043231), cytoplasm (GO:0005737), membrane (GO:0016020), and nucleus (GO:0005634) (Figure 6).

## 3. Discussion

Novel substances derived from animals, plants, and microorganisms with therapeutic application potential have been a major source of lead compounds for the pharmaceutical industry. In the drug discovery’s beginnings, more than 80% of the molecules were natural or nature-inspired compounds [32]. Classical natural product chemistry methodologies enabled a vast array of bioactive secondary metabolites from terrestrial and marine sources to be discovered.

Notably, the still unexplored marine environment and its unique biodiversity, along with venomous animal toxins, are a great source of new molecules, especially due to their high selectivity and efficacy when interacting with the targets, resulting in minimal side effects during disease treatment [33,34]. Some examples include Ziconotide, a peptide discovered in Conus magus, approved by U.S. Food and Drug Administration (FDA) for chronic pain treatment since 2004 [35]; and Exenatide, a synthetic form of a peptide found in the saliva of Heloderma suspectum that mimics the action of glucagon-like peptide-1, a new agent for type 2 diabetes mellitus treatment [36].

The peptide market is growing twice as fast as other drugs, suggesting they will soon receive even more merit. In view of increasing the *Tn*P dossier, using a patented peptide with immunomodulatory action in a murine model of multiple sclerosis [24], we recently demonstrated that *Tn*P possesses a wide therapeutic index without toxic effects on zebrafish. *Tn*P was safe in preclinical toxicological studies, crossing the blood–brain barrier without disturbing the normal architecture of the brain [15], which is fundamental once it is a candidate for the treatment of neuroinflammation, including neurodegenerative diseases.

The miRNAs world is a fascinating field that has been increasingly scrutinized in biological research. It occupies a prominent place at the avant-garde of genomics and provides a key and robust knowledge about gene regulation from lower to higher organisms. Here, we intended to gain insights into the regulation of gene expression through epigenetic processes potentially mediated by *Tn*P in inflamed zebrafish. *Tn*P induced the overexpression of known miRNAs, including miR-21, miR-122, miR-731, and miR-26. Our in silico analyses, looking for the putative mRNA targets of the miRNAs induced by *Tn*P, applied algorithms trained on a positive and a negative set of miRNA recognition elements (MREs) located in both the 3’-UTR and coding sequence (CDS) regions highly recommended to zebrafish trials that were adjusted in the threshold to avoid missing many authentic targets or including many false positives [37,38]. As a result, our analyses provided a relevant target gene set with enormous potential to be regulated by the *Tn*P-induced miRNAs investigated.

Interestingly, only 12 identified genes could be targeted by more than one miRNA (2.3%); it reveals they have very different targets, so a greater variety of functions and processes can be performed. The mutual gene set is very particular, and based on the UniProt [39] and GO annotations; the *skib* presents nucleotide and SMAD-binding functions, cartilage development, and dorsal–ventral pattern formation; SMADs are crucial for regulating cell development and growth. In zebrafish, the gene *fam217b* is expressed in the retina and other tissues, which can be correlated with its association with vision phenotype in humans, in addition to homeostasis and metabolism. The *manea* has mainly alpha-mannosidase activity, being expressed in the liver and other zebrafish tissues. Moreover, *rnd1b* main functions are GTPase activity, GTP binding, and protein kinase binding; it also contributes to cytoskeleton organization, cell shape, migration, and cell polarity. *smarca2* functions mainly as helicase, ligand ATP-binding, and nucleotide-binding. *nck1a* codes for a lipoprotein; it is expressed in granulocyte and other zebrafish tissues. *ppm1da* functions as hydrolase, protein phosphatase, and notably in metal ion binding using Mg^2+^ as a cofactor. *ogfr* presents opioid receptor activity and is expressed in embryos and 27 other tissues. *fhl3a* functions are related to metal ion binding, transcription coregulator activity, and actin cytoskeleton organization. *flj11011l* is predicted to have ubiquitin-conjugating enzyme activity involved in cellular protein metabolic process and cellular response to misfolded protein. *slc6a2* functions are not well characterized, but they may be related to transport, symport, and metal-binding. Finally, *igf2a* presents growth factor, hormone, and protein serine/threonine kinase activator activity and works in insulin-like growth factor receptor binding. It is also involved in many biological processes, such as notochord development, regulation of T cell proliferation, MAPK cascade, transcription, vascular endothelial cell proliferation, muscle cell differentiation, somitogenesis, and angiogenesis.

Our data show that miRNAs overexpressed by *Tn*P can regulate many genes in one pathway or even multiple cross-pathways, generating a tremendous impact on a complex regulatory network and, consequently, the magnitude of inflammation [40]. We observed that all miRNAs controlled many genes responsible for developmental and cellular processes and regulation of gene expression. These targeted genes can be related to the larval stage of zebrafish, a decisive phase in which the development of the main organs and tissues is completed. According to Vauti et al. [41], 87% of zebrafish genes are expressed in the first six days of development and only 13% in the second to fourth weeks.

However, the genes responsible for the regulation of transcription and DNA-template were only targeted by miR-26. Other recurrent target genes for all miRNAs were those associated with metabolic processes and stimulus response, consistent with LPS stimulation and treatment with *Tn*P. Several genes are expected to respond to antigen elicitation and to play their role in metabolizing both LPS and the therapeutic agent, i.e., *Tn*P.

Additionally, we have shown that genes related to neurogenesis and nervous system development functions were common through enrichment analysis for all miRNAs. We consider this a remarkable finding since our group has already proved the efficacy of the candidate peptide to treat EAE, a mouse model of MS. MS is a chronic, autoimmune disorder of the CNS leading to demyelination and neuronal loss associated with progressive neurological disability. Experimentally, *Tn*P led to accelerated remyelination in a cuprizone model of demyelination, validating *Tn*P as a very active anti-inflammatory and pro-remyelinating new peptide [24].

Among the zebrafish *Tn*P-induced miRNA targets related to the nervous system, we found that *sec63* and *myrf* are precisely involved in CNS myelination and analogous functions and *omgb* in neuron projection regeneration. Corroborating this evidence, orthologous to human myelin regulatory factor (*myrf*) constitutes a transcription factor precursor that explicitly activates transcription of CNS myelin genes, playing a central role in oligodendrocyte maturation and CNS myelination [42]. Moreover, human ortholog to oligodendrocyte myelin glycoprotein (*omgb*) works as a cell adhesion molecule contributing to the interactive process required for myelination [39]. In addition, Nohra et al. [43] brought to light that *rgma* (repulsive guidance molecule BMP co-receptor) ortholog gene is implicated in MS; in humans, this gene regulates cephalic neural tube closure, inhibits neurite outgrowth and cortical neuron branching, and is involved in the formation of mature synapses.

However, in general, most of these predicted nervous system-specific genes have DNA-binding transcription factor activity and additionally regulate, for example, neuron differentiation (*mosmob*; *acsl3b*; *draxin*), axon guidance (*lrrc15*), oligodendrocyte development (*prdm8b*), cerebellum development (*bmi1a*), brain development (*sez6l2*), neuron projection extension (*tnn*; *gpm6bb*), cell migration in the hindbrain and axis elongation (*dvl2*), and axonogenesis (*col6a2*; *ephb2b*) [42].

Unfortunately, not all predicted genes have reasonable inferences about their functions. Consequently, we used human orthologs to obtain clues about their roles. The *grna* ortholog gene is implicated in dementia, neurodegenerative disease, and neuronal ceroid lipofuscinosis 11; *gsk3ba* in Alzheimer’s disease, amyotrophic lateral sclerosis, bipolar disorder, and schizophrenia; *fzd3a* in Williams–Beuren syndrome and schizophrenia; *zc4h2* in Miles–Carpenter syndrome, a neuronal retardation disease; *neurod2* in early infantile epileptic encephalopathy; and *nrsn1l* in memory consolidation [42].

The miRNAs have not only been linked to the development of immune cells but also to infection or inflammation [44]. There is increasing evidence that they have important roles in regulating innate immune responses, the first line of defense to bacteria, viruses, and other pathogens. Some of these genes have been identified as targets of miRNAs induced by *Tn*P (Supplementary Table S1). We closely inspected this functional class because it represents the induced process in zebrafish for evaluating *Tn*P’s anti-inflammatory capacity.

Among these genes, we highlight *il17a*/*f2*, predicted to have cytokine activity in the zebrafish gill, intestine, and kidney. Its human orthologs, IL-17A and IL-17F, are implicated in autoimmune disease, chronic asthma, chronic mucocutaneous candidiasis, rhinitis, and MS. The *nlrc6*, orthologous to NLRP13, NLRP14, and NLRP2, is involved in intracellular signal transduction of the inflammasome complex. The *plcd4d* with phosphatidylinositol phospholipase C activity presents the human orthologous phospholipase C delta 4. The gene *pla2g6*, orthologous to human phospholipase A2 group VI, with predicted hydrolase activity, is involved in the cerebral lipid catabolic process. *pik3r3b* is predicted to have 1-phosphatidylinositol-3-kinase regulator activity in the immature eye, nervous system, neural tube, periderm, and somite. Two genes, *si:dkey-22i16.9* and *rnasel3*, are predicted to be involved in the immune response against LPS Gram-negative and Gram-positive bacteria, along with regulation of T cell activation. The human ortholog of *rnasel3* is implicated in multiple neurodegenerative diseases.

Similarly, *ifit15* might be involved in defense response to viruses. The genes *foxn1*, *zbtb11*, *smad1*, *klf3*, and *relb*, for instance, exhibit DNA-binding transcription factor activity. Specifically, *foxn1* is also involved in thymus development, where it is expressed, and its human ortholog is implicated in T cell immunodeficiency, while *relb* human ortholog is implicated in breast cancer and immunodeficiency. The *rag2* is strongly involved in immune system development; its human ortholog is linked to Omenn syndrome and severe combined immunodeficiency. Although they have their peculiarities, all of them are involved in innate immune response, and most of them have signaling receptor binding activity.

In addition, we found that the *si:dkey-42l23.7* can be predicted to have G protein-coupled and complement receptor activity. Diseases associated with its human orthologous *GPR33* (G protein-coupled receptor 33) include epidermolysis bullosa simplex with nail dystrophy and multiple epiphyseal dysplasia [39]. On the other hand, *relb* (v-rel avian reticuloendotheliosis viral oncogene homolog B) has transcription factor regulatory activity and chromatin binding activity. It is also involved in the innate immune response. Its human ortholog is also implicated in response to cytokine and associated with rheumatoid arthritis (RA). Among the ortholog-related pathways is the glial cell line-derived neurotrophic factor (GDNF)-family of ligands and receptor interactions and the immune response IL-23 signaling pathway. In murine models, *relb*, a component of the NF-kappaB complex of transcription factors, is a critical regulator of dendritic cells (DCs) differentiation; the lack of *relb* impairs DCs derived from bone marrow both in number and function. Zanetti et al. [45] used a *relb* –/– mice to study the antigen-presenting cells (APC) function of residual DCs in the presentation of soluble antigen and cross-priming; they found that mice DCs are profoundly deficient in their ability to both prime and cross-prime T cell responses, concluding that *relb* is involved in regulating the APC function of DCs in vivo.

These findings serve to help understand the effect of *Tn*P on the EAE model, in which we observed the activation of regulatory cells [24]. Female WT mice that were actively induced with MOG35-55 and treated with *Tn*P at 3 mg·kg^−1^ for 7 consecutive days showed increased expression of PDL-1 and PDL-2 in plasmacytoid DCs (CD11cintB220high) involved in the expansion of MOG35-55-specific Treg cells that inhibit EAE. *Tn*P inhibited the function of pathogenic CD4^+^ T cells and induced the development of Foxp3+ Treg, without the development of Th2 or CD5^+^ CD1d^+^ Breg cells.

Still, the other target gene involved in the inflammatory response is *ptgs2b*. It exhibits peroxidase activity and is involved in the cellular response to organic cyclic compounds (such as *Tn*P). Its human ortholog is implicated in several diseases, including Barrett’s esophagus, artery disease, and arthritis; it is also involved in the prostaglandin biosynthesis pathway, which is part of lipid metabolism. Prostaglandin synthases, prostanoids, and their receptors have their expression altered in zebrafish gonads, suggesting *ptgs2b* participates in ovulation and juvenile ovary to testis transition in zebrafish [46].

Furthermore, in our in silico analyzes, the molecular functions associated with binding, from small molecules binding to DNA and protein binding, were noticeable among the most prominent enriched terms. These gene products present a great affinity to other molecules and act as ligands, which provide a selective, non-covalent, often stoichiometric interaction of a molecule with one or more specific sites on another molecule. Some targets of all miRNAs presented catalytic, hydrolase, kinase, and transferase activities. This is expected since RNAs per se are intensely involved in such reactions.

In addition, miR-21, miR-731, and miR-26 were involved in molecular functions, such as transcriptional regulatory activity, corroborating the analysis of the PANTHER protein class, which showed that predicted genes code mainly for DNA-binding transcription factors and specific transcriptional regulatory proteins (Figure 5). Those contributions are another layer of the genetic expression multiplexed regulation. The miRNAs take part in the post-transcriptional regulation, meaning they regulate mRNA transcripts already synthesized [47], but what we have shown here is that their targets are also strongly related to expression regulation at the transcriptional level. This kind of control is performed, for example, by transcription factors, which stimulate genes to be transcribed and which are ubiquitous miRNA-controlled proteins; to a lesser extent miR-122, whose target genes presented different protein classes, such as GTPase-activating protein, kinase, G-protein modulator, and scaffold/adaptor protein.

A notable transcription factor that can illustrate the interconnections between miRNAs and proteins involved in genomic expression regulation is the aryl hydrocarbon receptor (AhR), a protein that belongs to the basic helix-loop-helix (bHLH)-PER-ARNT-SIM family [48]. AhR has been extensively investigated as a classic environmentally responsive sensor triggered by a broad range of exogenous and endogenous compounds, with deep characterization over the ligands 2,3,7,8-Tetrachlorodibenzo-p-dioxin (TCDD) and benzo[a]pyrene (BaP) [49,50]. The AhR-ARNT complex enables receptor binding to cognate DNA consensus sequences, promoting their transcription.

However, more recently, AhR has been related to inflammatory response, oxidative stress, apoptosis, immune regulation, and tumorigenesis through mechanisms involving miRNAs, where activated AhR increases the expression of several miRNAs [51,52]. On the other hand, miRNAs can also regulate AhR and other proteins in the complex (e.g., ARNT). For example, according to TargetScan analysis, AhR 3′-UTR contains the binding site for miR-124, which is highly conserved among humans, mice, and most species. Experimental findings confirm that miR-124 may affect neuroblastoma cell differentiation by targeting AhR [53], promotes the intestinal inflammation in Crohn’s disease [54], and regulates cellular inflammatory response through negatively regulating AhR expression in chronic rhinosinusitis [51].

Overall, comparing all the predicted targets of the four miRNAs, some genes participate simultaneously in a high number of different biological processes and molecular functions, meaning they might have a broader range of functionalities. In this context, we highlight a few genes (present in >7 enriched GO terms) from the terms discussed before, such as *foxo3a* (forkhead box O3A), *rbpjb* (recombination signal binding protein for immunoglobulin kappa J region b), *rxrbb* (retinoid × receptor, beta b), *tyrobp* (transmembrane immune signaling adaptor TYROBP), *hes6* (hes family bHLH transcription factor 6), *zic5* (zic family member 5), *smad1* (SMAD family member 1), *e2f7* (E2F transcription factor 7), and *npas4a* (neuronal PAS domain protein 4a), among others. Again, only the miR-122 predicted target genes were individually involved in fewer processes, especially compared with miR-21 and miR-26, whose targets seem to be involved in various molecular and biological activities.

The miR-122 targets were the only ones not profoundly involved in transcription regulator activity and not participating concomitantly in many functions; the likely reason for that performance is because miR-122 is a tissue-specific miRNA. It is highly expressed in the liver, and its mature sequence is completely conserved in the vertebrate lineage [55,56], leading to the understanding that its roles are connected to more specific purposes. Chin Tai and Freeman [18] elaborated in a review the miRNA mechanisms of action using zebrafish, demonstrating that miR-122 may be involved in the toxicological response to environmental contaminants, such as particulate matter, nanoparticles, CuCl_2_, fluoxetine, valproic acid, tamoxifen, and acetaminophen. Pasqualotto et al. [57] have shown that chronic zebrafish exposure to ethanol leads to altered hepatic expression of miR-122. Additionally, Wu et al. [58] registered an overexpression of miR-122 in zebrafish liver (ZFL) cells after LPS stimulation, confirming that it plays an important role in zebrafish immunology.

Moreover, there is a confirmed involvement of miR-21 in the inflammatory response following infection with *Aeromonas hydrophila* and LPS stimulation in grass carp, where downregulation of miR-21 promotes proinflammatory cytokine expression [59]. In zebrafish, miR-21 has been reported by Wu et al. [60] to impact cardiac valvulogenesis in embryos, and the authors applied a quantitative proteomic strategy to identify the global profile of miR-21-regulated proteins; 251 proteins were dysregulated after miRNA knockdown, suggesting that it possesses pleiotropic functions and may be involved in diverse cellular pathways. Moreover, the involvement of miR-21 with environmental toxicity was observed [18].

When it comes to miR-731, it has been demonstrated to play a role in zebrafish liver and exocrine pancreas development by directly targeting *dkk3b*, affecting digestive organ development [61]. It was also found to mediate chlorpyrifos-induced head kidney injury in common carp by targeting TLR and apoptosis pathways [62].

Simultaneously, scientific information exhibits that zebrafish miR-26 acts in regulating silica nanoparticles, atrazine, and fluoxetine toxic response [18]. Furthermore, overexpression of miR-26a adversely affected physiological angiogenesis by impairing the caudal vein plexus (CVP) formation, a BMP-responsive region in zebrafish—an effect rescued by ectopic SMAD1 expression. In mice, miR-26a overexpression inhibited EC SMAD1 expression and exercise-induced angiogenesis [63]. Still, in mice, miR-26 suppresses adipocyte progenitor differentiation and fat production by targeting *Fbxl19*, revealing a novel pathway in adipose tissue formation and a new potential therapeutic target for obesity [64]. Finally, we identified miR-26a as one key regulator of *Tn*P anti-inflammatory effects. Zebrafish in which miR-26a was knocked down sustained an elevated number of neutrophils in the inflamed wound, demonstrating that the absence of the miR-26a promoted a failure in the regulatory capacity of *Tn*P [65].

The over-representation analysis of GO terms provided an efficacious manner to understand the biological activities in which the target gene products participate. Some informative roles are related to gene expression regulation, neurogenesis, DNA binding, transcription regulation, immune system process, and inflammatory response. However, other functional categories, such as cellular process, biological regulation, and metabolic process, were well represented. The high similarity in the over-represented GO terms among the four miRNA targets suggests that they are involved in similar processes, which may be due to the concordant experimental condition (LPS-stimulated larvae followed by *Tn*P treatment). However, the predicted genes separately were very distinct to each one of the miRNAs investigated. Furthermore, despite computational studies, specific miRNA targets must be validated by experimental assays to verify their roles.

Although we faced some limitations starting the data collection, such as scarce availability of online predictors powered with zebrafish data compared with long-established models and fewer miRNAs annotated (in the miRBase, *Homo sapiens* and *Mus musculus* exhibit approximately 7 and 5 times more mature miRNAs annotated than *Danio rerio*, respectively); still this study is genuinely relevant, primarily because it focuses on a potential new drug resulting from marine biodiversity, one of the main interests of the peptide market and pharmaceutical industry. Additionally, these findings add to the previous results about *Tn*P anti-inflammatory evidence in murine models and its preclinical safety, which together make up the ever-expanding *Tn*P report.

Both the in silico analysis and the use of zebrafish, considered an alternative animal model, bring many advantages in the early drug discovery stage and screening for therapeutic candidates, meeting the 3Rs (reduction, refinement, and replacement) philosophy of experimental biology. The present study provides valuable information concerning the gene expression regulation through epigenetic mechanisms driven by miR-21, miR-122, miR-731, and miR-26 in zebrafish treated with the anti-inflammatory pre-drug *Tn*P. Ultimately, these findings not only provide new insights into the miRNAs’ mode of action but also raise hope for miRNA-driven *Tn*P therapy and may direct further experimental investigations.

## 4. Methods

### 4.1. LPS Challenge and TnP Treatment

The experimental procedures were carried out with 72 h post-fertilization (hpf) wildtype (WT) AB strain zebrafish larvae originally from the International Zebrafish Resource Center (Eugene, OR, USA), kept in plastic dishes (100 × 20 mm) containing E2 0.5× medium (7.5 mM KH_2_PO_4_, 2.5 mM Na_2_HPO_4_, 15 mM NaCl, 0.5 mM KCl, 1 mM MgSO_4_7H_2_O, 1 mM CaCl_2_2H_2_O, 0.7 mM NaHCO_3_) and incubated at 28 °C.

Briefly, after tailfin transection, injury larvae were stimulated with LPS from Salmonella abortus (L5886, Sigma) at 100 µg·mL-1 for 1 h at 28 °C and thereafter treated with TnP (#P13821401, GenScript, Piscataway, NJ, USA) at 0.01 μM by immersion for another 1 h at 28 °C. All animal husbandry and experiments were carried out in full accordance with the laws of experimental animal welfare and detailed by Falcao et al. (2021) [65].

### 4.2. Selection of dre-miRNAs for the In Silico Target Prediction Analysis

RNA-seq libraries from *Tn*P-treated LPS-inflamed zebrafish larvae, with high RNA integrity number (RIN) (range, 7.8–10), were generated using the Ion Total RNA-Seq Kit v2. The samples were sequenced using Illumina NovaSeq and HiSeq platforms with paired-end 150 bp (PE 150) sequencing strategy, according to Falcao et al. 2021 [65].

Among the genes identified in LPS-challenged zebrafish under *Tn*P treatment, the four known dre-miRNAs that showed the highest expression levels were selected: miR-21 (5′-UAGCUUAUCAGACUGGUGUUGGC-3′); miR-122 (5′-UGGAGUGUGACAAUGGUGUUUG-3′); miR-731 (5′-AAUGACACGUUUUCUCCCGGAUCG-3′); miR-26a (5′-UUCAAGUAAUCCAGGAUAGGCU-3′); and miR-26b (5′-UUCAAGUAAUCCAGGAUAGGUU-3′) (www.mirbase.org, accessed on 29 April 2021).

### 4.3. Data Sources

MiRBase is the leading data repository for storing miRNA information from different experimental and computational discoveries and various species, including zebrafish [66]. Reference zebrafish miRNAs (dre-miR) along with 373 mature miRNAs were taken from miRBase 21 (http://www.mirbase.org/, accessed on 15 September 2020) for the analysis.

### 4.4. In Silico Target Prediction Analysis

Two bioinformatics software packages, DIANA microT-CDS v. 5.0 (http://diana.imis.athena-innovation.gr/DianaTools/index.php?r=microT_CDS/index, Thessaly, Greece; accessed on 15 September 2020) [37,67] and TargetScanFish v. 6.2 (http://www.targetscan.org/fish_62/, Cambridge, MA, USA; accessed on 15 September 2020) [38] were programmed to predict the putative miRNA:mRNA interaction sites applying: the miRNA sequence; the 3′-UTR sequence; the Watson–Crick base pairing in 5′ end of the miRNA, called *seed* region; the free energy expressed in kcal/mol, a measure of binding stability; the 3′ region of the miRNA that also have a Watson–Crick base pairing with the mRNA; the level of conservation of this interaction between species; other regions with Watson–Crick base pairing in the 5′-UTR, open read frame (ORF) and CDS; and other factors unrelated to the Watson–Crick base pairing that can affect the miRNA action, called context. Only predictions with a miTG score > 0.7 and score + context < −0.2, respectively, were considered as effective miRNA targets.

The two prediction lists were overlapped, and a set of mutually predicted targets was generated for all miRNAs. The predicted genes that were retired from the ensemble were deducted from each software prediction list.

### 4.5. Gene Ontology Enrichment

We performed gene ontology (GO) enrichment analysis to identify biological process, molecular function, and cellular component terms (the GO current release, 1 February 2021, presents 44.085 terms) (http://geneontology.org/, accessed on 10 October 2020) [68], using either the GO database or the PANTHER (Protein Analysis Through Evolutionary Relationships) Classification System, whose annotations provide a set of terms associated with the imputed genes through a hierarchical list of determining functional GO terms (http://www.pantherdb.org/, accessed on 10 October 2020),according to Mi et al. [69,70]. The PANTHER classification system is a resource for the evolutionary and functional classification of protein-coding genes from all domains of life. It is an extensive curated biological database of gene and protein families, and its most important application is to accurately infer the function of uncharacterized genes from any organism based on their evolutionary relationships to genes with known functions. PANTHER is part of the Gene Ontology Reference Genome Project [71].

## Supplementary Materials

The following are available online at https://www.mdpi.com/article/10.3390/ijms22137117/s1, Table S1: List of predicted target genes of *Tn*P-upregulated *Danio rerio* miR-21, miR-122, miR-731, and miR-26 by Diana microT-CDS (miTG score > 0.7) and TargetScanFish (score + context < −0.2).

## References

[1] Jirtle R.L., Skinner M.K. (2007). Environmental epigenomics and disease susceptibility. Nat. Rev. Genet..

[2] Lee R.C., Feinbaum R.L., Ambros V. (1993). The *C. elegans* heterochronic gene lin-4 encodes small RNAs with antisense complementarity to lin-14. Cell.

[3] Bhaskaran M., Mohan M. (2014). MicroRNAs: History, Biogenesis, and Their Evolving Role in Animal Development and Disease. Vet. Pathol..

[4] Lewis B.P., Burge C.B., Bartel D.P. (2005). Conserved seed pairing, often flanked by adenosines, indicates that thousands of human genes are microRNA targets. Cell.

[5] Bartel D.P. (2018). Metazoan MicroRNAs. Cell.

[6] Slota J.A., Booth A.S. (2019). MicroRNAs in Neuroinflammation: Implications in Disease Pathogenesis, Biomarker Discovery and Therapeutic Applications. Noncoding RNA.

[7] Selbach M., Schwanhausser B., Thierfelder N., Fang Z., Khanin R., Rajewsky N. (2008). Widespread changes in protein synthesis induced by microRNAs. Nature.

[8] Friedman R.C., Farh K.K., Burge C.B., Bartel D.P. (2009). Most mammalian mRNAs are conserved targets of microRNAs. Genome Res..

[9] Thompson F., de Oliveira B.C., Cordeiro M.C., Masi B.P., Rangel T.P., Paz P., Freitas T., Lopes G., Silva B.S., Cabral A. (2020). Severe impacts of the Brumadinho dam failure (Minas Gerais, Brazil) on the water quality of the Paraopeba River. Sci. Total Environ..

[10] Lopes-Ferreira M., Falcão M.A.P., Disner G.R., Lima C., Silva C.V.R.D., Queiroz L.G., Gomes L.E.T. (2021). O modelo Zebrafish e sua contribuição ao meio ambiente. Recurso Água: Tecnologias e Pesquisas Para o uso e a Conservação de Ecossistemas Aquáticos.

[11] Disner G.R., Falcao M.A.P., Andrade-Barros A.I., Leite-Santos N.V., Soares A.B.S., Marcolino-Souza M., Gomes K.S., Lima C., Lopes-Ferreira M. (2020). The toxic effects of glyphosate, chlorpyrifos, abamectin, and 2,4-D on animal models: A systematic review of Brazilian studies. Integr. Environ. Assess. Manag..

[12] MacRae C.A., Peterson R.T. (2015). Zebrafish as tools for drug discovery. Nat. Rev. Drug. Discov..

[13] Garcia G.R., Noyes P.D., Tanguay R.L. (2016). Advancements in zebrafish applications for 21st century toxicology. Pharmacol. Ther..

[14] Zakaria Z.Z., Benslimane F.M., Nasrallah G.K., Shurbaji S., Younes N.N., Mraiche F., Da’as S.I., Yalcin H.C. (2018). Using Zebrafish for Investigating the Molecular Mechanisms of Drug-Induced Cardiotoxicity. Biomed. Res. Int..

[15] Batista-Filho J., Falcão M.A.P., Maleski A.L.A., Soares A.B.S., Balan-Lima L., Disner G.R., Lima C., Lopes-Ferreira M. (2021). Early preclinical screening using zebrafish (*Danio rerio*) reveals the safety of the candidate anti-inflammatory therapeutic agent TnP. Toxicol. Rep..

[16] Bizuayehu T.T., Babiak I. (2014). MicroRNA in teleost fish. Genome Biol. Evol..

[17] Desvignes T., Beam M.J., Batzel P., Sydes J., Postlethwait J.H. (2014). Expanding the annotation of zebrafish microRNAs based on small RNA sequencing. Gene.

[18] Chin Tai J.K.A., Freeman J.L. (2020). Zebrafish as an integrative vertebrate model to identify miRNA mechanisms regulating toxicity. Toxicol. Rep..

[19] Gurol T., Zhou W., Deng Q. (2016). MicroRNAs in neutrophils: Potential next generation therapeutics for inflammatory ailments. Immunol. Rev..

[20] Hsu A.Y., Wang D., Gurol T., Zhou W., Zhu X., Lu H., Deng Q. (2017). Overexpression of microRNA-722 fine-tunes neutrophilic inflammation by inhibiting Rac2 in zebrafish. Dis. Model. Mech..

[21] Hsu A.Y., Wang D., Liu S., Lu J., Syahirah R., Bennin D.A., Huttenlocher A., Umulis D.M., Wan J., Deng Q. (2019). Phenotypical microRNA screen reveals a noncanonical role of CDK2 in regulating neutrophil migration. Proc. Natl. Acad. Sci. USA.

[22] Ji C., Guo X., Ren J., Zu Y., Li W., Zhang Q. (2019). Transcriptomic analysis of microRNAs-mRNAs regulating innate immune response of zebrafish larvae against *Vibrio parahaemolyticus* infection. Fish Shellfish Immunol..

[23] Lopes-Ferreira M.V.A., Lima C., Pimenta D.C., Conceição K., Demasi M., Portaro F.C.V. (2019). Peptídeos Cíclicos Anti-Inflamatórios e Anti-Alérgicos.

[24] Komegae E.N., Souza T.A., Grund L.Z., Lima C., Lopes-Ferreira M. (2017). Multiple functional therapeutic effects of TnP: A small stable synthetic peptide derived from fish venom in a mouse model of multiple sclerosis. PLoS ONE.

[25] Lopes-Ferreira M., Lima C. (2021). Personal Communication.

[26] Peterson S.M., Thompson J.A., Ufkin M.L., Sathyanarayana P., Liaw L., Congdon C.B. (2014). Common features of microRNA target prediction tools. Front. Genet..

[27] Riffo-Campos Á.L., Riquelme I., Brebi-Mieville P. (2016). Tools for Sequence-Based miRNA Target Prediction: What to Choose?. Int. J. Mol. Sci..

[28] Huang H.Y., Lin Y.C., Li J., Huang K.Y., Shrestha S., Hong H.C., Tang Y., Chen Y.G., Jin C.N., Yu Y. (2020). miRTarBase 2020: Updates to the experimentally validated microRNA-target interaction database. Nucleic Acids Res..

[29] Rajewsky N. (2006). microRNA target predictions in animals. Nat. Genet..

[30] Griffiths-Jones S., Saini H.K., van Dongen S., Enright A.J. (2008). miRBase: Tools for microRNA genomics. Nucleic Acids Res..

[31] Forn-Cuní G., Varela M., Pereiro P., Novoa B., Figueras A. (2017). Conserved gene regulation during acute inflammation between zebrafish and mammals. Sci. Rep..

[32] Harvey A., Edrada-Ebel R., Quinn R. (2015). The re-emergence of natural products for drug discovery in the genomics era. Nat. Rev. Drug Discov..

[33] Akondi K.B., Muttenthaler M., Dutertre S., Kaas Q., Craik D.J., Lewis R.J., Alewood P.F. (2014). Discovery, synthesis, and structure-activity relationships of conotoxins. Chem. Rev..

[34] Mahadevappa R., Ma R., Kwok H.F. (2017). Venom Peptides: Improving Specificity in Cancer Therapy. Trends Cancer.

[35] Pope J.E., Deer T.R. (2013). Ziconotide: A clinical update and pharmacologic review. Exp. Opin. Pharmacother..

[36] Furman B.L. (2012). The development of Byetta (exenatide) from the venom of the *Gila monster* as an anti-diabetic agent. Toxicon.

[37] Paraskevopoulou M.D., Georgakilas G., Kostoulas N., Vlachos I.S., Vergoulis T., Reczko M., Filippidis C., Dalamagas T., Hatzigeorgiou A.G. (2013). DIANA-microT web server v5.0: Service integration into miRNA functional analysis workflows. Nucl. Acids Res..

[38] Lewis B.P., Shih I.H., Jones-Rhoades M.W., Bartel D.P. (2003). Prediction of mammalian MicroRNA targets. Cell.

[39] UniProt Knowledgebase The UniProt Consortium: European Bioinformatics Institute (EMBL-EBI), SIB Swiss Institute of Bioinformatics, and Protein Information Resource (PIR). https://www.uniprot.org/.

[40] Thomas P.D., Dessimoz C., Škunca N. (2017). The Gene Ontology and the Meaning of Biological Function. The Gene Ontology Handbook. Methods in Molecular Biology.

[41] Vauti F., Stegemann L.A., Vögele V., Köster R.W. (2020). All-age whole mount in situ hybridization to reveal larval and juvenile expression patterns in zebrafish. PLoS ONE.

[42] Zebrafish Information Network (ZFIN) University of Oregon, Eugene, OR 97403-5274. http://zfin.org/.

[43] Nohra R., Beyeen A.D., Guo J.P., Khademi M., Sundqvist E., Hedreul M.T., Sellebjerg F., Smestad C., Oturai A.B., Harbo H.F. (2010). RGMA and IL21R show association with experimental inflammation and multiple sclerosis. Genes Immun..

[44] Andreassen R., Høyheim B. (2017). miRNAs associated with immune response in teleost fish. Dev. Comp. Immunol..

[45] Zanetti M., Castiglioni P., Schoenberger S., Gerloni M. (2003). The role of relB in regulating the adaptive immune response. Ann. N.Y. Acad. Sci..

[46] Pradhan A., Olsson P.E. (2014). Juvenile Ovary to Testis Transition in Zebrafish Involves Inhibition of Ptges. Biology Reprod..

[47] Bethune J., Artus-Revel C.G., Filipowicz W. (2012). Kinetic analysis reveals successive steps leading to miRNA-mediated silencing in mammalian cells. EMBO Rep..

[48] Gutiérrez-Vázquez C., Quintana F.J. (2018). Regulation of the Immune Response by the Aryl Hydrocarbon Receptor. Immunity.

[49] Hanieh H., Mohafez O., Hairul-Islam V.I., Alzahrani A., Bani Ismail M., Thirugnanasambantham K. (2016). Novel Aryl Hydrocarbon Receptor Agonist Suppresses Migration and Invasion of Breast Cancer Cells. PLoS ONE.

[50] Lv Q., Shi C., Qiao S., Cao N., Guan C., Dai Y., Wei Z. (2018). Alpinetin exerts anti-colitis efficacy by activating AhR, regulating miR-302/DNMT-1/CREB signals, and therefore promoting Treg differentiation. Cell Death Dis..

[51] Liu C.C., Xia M., Zhang Y.J., Jin P., Zhao L., Zhang J., Li T., Zhou X.M., Tu Y.Y., Kong F. (2018). Micro124-mediated AHR expression regulates the inflammatory response of chronic rhinosinusitis (CRS) with nasal polyps. Biochem. Biophys. Res. Commun..

[52] Rogers S., Souza A.R., Zago M., Iu M., Guerrina N., Gomez A., Matthews J., Baglole C.J. (2017). Aryl hydrocarbon receptor (AhR)-dependent regulation of pulmonary miRNA by chronic cigarette smoke exposure. Sci. Rep..

[53] Huang T.-C., Chang H.-Y., Chen C.-Y., Wu P.-Y., Lee H., Liao Y.-F., Hsu W.-M., Huang H.-C., Juan H.-F. (2011). Silencing of miR-124 induces neuroblastoma SK-N-SH cell differentiation, cell cycle arrest and apoptosis through promoting AHR. FEBS Lett..

[54] Zhao Y., Ma T., Chen W., Chen Y., Li M., Ren L., Chen J., Cao R., Feng Y., Zhang H. (2016). MicroRNA-124 Promotes Intestinal Inflammation by Targeting Aryl Hydrocarbon Receptor in Crohn’s Disease. J. Crohns. Colitis.

[55] Wienholds E., Kloosterman W.P., Miska E., Alvarez-Saavedra E., Berezikov E., de Bruijn E., Horvitz H.R., Kauppinen S., Plasterk R.H. (2005). MicroRNA expression in zebrafish embryonic development. Science.

[56] Jopling C. (2012). Liver-specific microRNA-122: Biogenesis and function. RNA Biol..

[57] Pasqualotto A., Ayres R., Longo L., Lima D.D., Oliveira D.L., Alvares-da-Silva M.R., Silveira T.R., Uribe-Cruz C. (2021). Chronic exposure to ethanol alters the expression of miR-155, miR-122 and miR-217 in alcoholic liver disease in an adult zebrafish model. Biomarkers.

[58] Wu T.-H., Pan C.-Y., Lin M.-C., Hsieh J.-C., Hui C.-F., Chen J.-Y. (2012). In vivo screening of zebrafish microRNA responses to bacterial infection and their possible roles in regulating immune response genes after lipopolysaccharide stimulation. Fish Physiol. Biochem..

[59] Tao L., Xu X., Fang Y., Wang A., Zhou F., Shen Y., Li J. (2019). miR-21 targets jnk and ccr7 to modulate the inflammatory response of grass carp following bacterial infection. Fish Shellfish Immunol..

[60] Wu Y., Lou Q.Y., Ge F., Xiong Q. (2017). Quantitative Proteomics Analysis Reveals Novel Targets of miR-21 in Zebrafish Embryos. Sci. Rep..

[61] Huang Y., Huang C.-X., Wang W.-F., Liu H., Wang H.-L. (2020). Zebrafish miR-462-731 is required for digestive organ development. Comp. Biochem. Physiol. Part D Genom. Proteom..

[62] Liu Q., Yang J., Gong Y., Cai J., Zhang Z. (2019). Role of miR-731 and miR-2188-3p in mediating chlorpyrifos induced head kidney injury in common carp via targeting TLR and apoptosis pathways. Aquat. Toxicol..

[63] Icli B., Wara A.K., Moslehi J., Sun X., Plovie E., Cahill M., Marchini J.F., Schissler A., Padera R.F., Shi J. (2013). MicroRNA-26a regulates pathological and physiological angiogenesis by targeting BMP/SMAD1 signaling. Circ. Res..

[64] Acharya A., Berry D.C., Zhang H., Jiang Y., Jones B.T., Hammer R.E., Graff J.M., Mendell J.T. (2019). miR-26 suppresses adipocyte progenitor differentiation and fat production by targeting Fbxl19. Genes Dev..

[65] Falcao M.A.P., Walker C.I.B., Disner G.R., Batista-Filho J., Soares A.B.S., Balan-Lima L., Lima C., Lopes-Ferreira M. Knockdown of miR-26a in Zebrafish Leads to Impairment of the Anti-Inflammatory Function of TnP in the Control of Neutrophilia. Fish Shellfish Immunol..

[66] Griffiths-Jones S., Grocock R.J., van Dongen S., Bateman A., Enright A.J. (2006). miRBase: microRNA sequences, targets and gene nomenclature. Nucl. Acids Res..

[67] Reczko M., Maragkakis M., Alexiou P., Grosse I., Hatzigeorgiou A.G. (2012). Functional microRNA targets in protein coding sequences. Bioinformatics.

[68] The Gene Ontology Consortium (2017). Expansion of the Gene Ontology knowledgebase and resources. Nucleic Acids Res..

[69] Mi H., Muruganujan A., Huang X., Ebert D., Mills C., Guo X., Thomas P.D. (2019). Protocol Update for large-scale genome and gene function analysis with the PANTHER classification system (v.14.0). Nat. Protoc..

[70] Mi H., Muruganujan A., Ebert D., Huang X., Thomas P.D. (2019). PANTHER version 14: More genomes, a new PANTHER GO-slim and improvements in enrichment analysis tools. Nucleic Acids Res..

[71] Mi H., Muruganujan A., Casagrande J.T., Thomas P.D. (2013). Large-scale gene function analysis with the PANTHER classification system. Nat. Protoc..

