# Supplementary material for: In Silico Target Prediction of Overexpressed microRNAs from LPS-Challenged Zebrafish (Danio rerio) Treated with the Novel Anti-Inflammatory Peptide TnP
Source: Int J Mol Sci. 2021 Jul 1;22(13):7117. doi: 10.3390/ijms22137117 (PMC8268205; doi:10.3390/ijms22137117)
Supplement: Supplementary file 1 [file ijms-22-07117-s001.zip › ijms-1160697-supplementary.pdf]

Supplementary Table S1. List of predicted target genes of *Tn*P-upregulated *Danio rerio* miR-21, miR-122, miR-731, and miR-26 by Diana microT-CDS (miTG score > 0.7) and TargetScanFish (score + context < -0.2).

miRNA	Ensembl Target Gene Id	Target gene name	Target Gene Synonyms	Description	DIANA microT-CDS miTG score	TargetScan Total context + score
miR-21	ENSDARG00000008034	*skib*	*fc17g02, skiB, wu:fc17g02, wu:fk79g04*	v-ski avian sarcoma viral oncogene homolog b	0.99	-0.35
miR-21	ENSDARG00000021443	*zfp36l1b*	*fc06f10, wu:fc06f10, zfp36l1l, zgc:77882*	zinc finger protein 36, C3H type-like 1b	0.98	-0.32
miR-21	ENSDARG00000001898	*manea*	*fi29h09, wu:fi29h09, zgc:92825*	mannosidase, endo-alpha	0.96	-0.60
miR-21	ENSDARG00000076566	*kank3*	*NBP, Numb-Binding Protein, si:dkeyp-104h9.6*	KN motif and ankyrin repeat domains 3	0.96	-0.21
miR-21	ENSDARG00000038845	*ldhd*	*wu:fi36b04, zgc:55447*	lactate dehydrogenase D	0.96	-0.65
miR-21	ENSDARG00000074812	*fhdc1*	*si:ch211-62k15.3*	formin homology domain containing 1	0.95	-0.42
miR-21	ENSDARG00000008363	*mcl1b*	*zgc:109763*	MCL1 apoptosis regulator, BCL2 family member b	0.94	-0.25
miR-21	ENSDARG00000045160	*msrb1b*	*sepx1b, zgc:92822*	methionine sulfoxide reductase B1b	0.94	-0.38
miR-21	ENSDARG00000045516	*itih2*	*id:ibd5015, wu:fb34d10, wu:fb96f01, zgc:56119*	inter-alpha-trypsin inhibitor heavy chain 2	0.94	-0.39
miR-21	ENSDARG00000007369	*tcf7l1b*	*tcf3b*	transcription factor 7 like 1b	0.94	-0.37
miR-21	ENSDARG00000070781	*gjd2b*	*cx35, cx35.1, cx35b, gja9*	gap junction protein delta 2b	0.93	-0.54
miR-21	ENSDARG00000052594	*nkiras2*	*zgc:92870*	NFKB inhibitor interacting Ras-like 2	0.92	-0.42
miR-21	ENSDARG00000004218	*rnd1b*	*Rnd1a, rnd1l, wu:fk50c04, zgc:153089*	Rho family GTPase 1b	0.90	-0.45
miR-21	ENSDARG00000073883	*clstn3*	*si:dkey-197a20.4*	calsyntenin 3	0.89	-0.38
miR-21	ENSDARG00000016835	*tcirg1a*	*si:dkey-9i23.9, tcirg1*	T cell immune regulator 1, ATPase H+ transporting V0 subunit a3a	0.89	-0.63
miR-21	ENSDARG00000020228	*usf2*	*fb12g11, si:dkey-211g8.3, wu:fb12g11*	upstream transcription factor 2, c-fos interacting	0.89	-0.31
miR-21	ENSDARG00000075714	*hoatz*	*C5H11orf88, si:dkey-27p23.3*	HOATZ cilia and flagella associated protein	0.88	-0.42
miR-21	ENSDARG00000023058	*foxo3a*	*fb50g02, fi33f07, wu:fb50g02, wu:fi33f07, zgc:92176*	forkhead box O3A	0.88	-0.26
miR-21	ENSDARG00000011879	*foxn1*	*nude, whnb*	forkhead box N1	0.87	-0.52
miR-21	ENSDARG00000005343	*ccdc85al*	*si:dkey-277e10.1, zgc:92780*	coiled-coil domain containing 85A, like	0.86	-0.38
miR-21	ENSDARG00000042029	*mbd3b*	*cb357, cb99, sb:cb357, sb:cb99, wu:fc41c05, zgc:111851*	methyl-CpG binding domain protein 3b	0.86	-0.34
miR-21	ENSDARG00000090072	*CU462878.2*	*KRTCAP3*	keratinocyte associated protein 3	0.85	-0.21
miR-21	ENSDARG00000075532	*slc4a11*	*si:dkey-12j14.4*	solute carrier family 4 member 11	0.85	-0.31
miR-21	ENSDARG00000036457	*cacng6a*	*si:dkey-234p10.2*	calcium channel, voltage-dependent, gamma subunit 6a	0.85	-0.36
miR-21	ENSDARG00000022768	*gid8a*	*zgc:73100*	GID complex subunit 8 homolog a	0.85	-0.40
miR-21	ENSDARG00000043482	*b3gnt3.1*	*zgc:136488, zgc:86586*	UDP-GlcNAc:betaGal beta-1,3-N-acetylglucosaminyltransferase 3, tandem duplicate 1	0.85	-0.38
miR-21	ENSDARG00000042368	*kif26aa*	*si:dkey-31d8.1*	kinesin family member 26Aa	0.85	-0.30
miR-21	ENSDARG00000008904	*smarca2*	*wu:fa56c07, wu:fi27f11, zgc:66238*	SWI/SNF related, matrix associated, actin dependent regulator of chromatin, subfamily a, member 2	0.84	-0.31
miR-21	ENSDARG00000074262	*nck1a*	*si:dkey-4m11.4, nck1*	NCK adaptor protein 1a	0.83	-0.30
miR-21	ENSDARG00000002013	*grb10a*	*grb10, zgc:91987*	growth factor receptor-bound protein 10a	0.83	-0.31
miR-21	ENSDARG00000052957	*plcd3a*	*DrPLC-delta 3A, zgc:158396*	phospholipase C, delta 3a	0.83	-0.29
miR-21	ENSDARG00000090914	*si:ch211-117k10.3*	*klf15l, AL929286.1*	*si:ch211-117k10.3*; orthologous to human KLF15 (Kruppel like factor 15)	0.83	-0.34
miR-21	ENSDARG00000076913	*eme1*	*fc30c07, wu:fc30c07*	essential meiotic structure-specific endonuclease 1	0.82	-0.34
miR-21	ENSDARG00000052091	*rbpjb*	*Su(H)2*	recombination signal binding protein for immunoglobulin kappa J region b	0.82	-0.23
miR-21	ENSDARG00000005943	*htra4*	*si:dkey-49o11.4*	HtrA serine peptidase 4	0.81	-0.31
miR-21	ENSDARG00000037421	*egr1*	*etID309970.14, krox-24, krox24, wu:fj64b05, wu:fq25f01*	early growth response 1	0.81	-0.22
miR-21	ENSDARG00000026236	*zgc:56585*	*zgc:77077*	*zgc:56585*; orthologous to human HPGD (15-hydroxyprostaglandin dehydrogenase).	0.81	-0.37
miR-21	ENSDARG00000088022	*mhc1zfa*	N/A	major histocompatibility complex class I ZFA	0.81	-0.24
miR-21	ENSDARG00000002006	*rxrbb*	*NR2B2-B, rxrd, unp286*	retinoid x receptor, beta b	0.81	-0.28
miR-21	ENSDARG00000039423	*AL1*	*si:ch211-217g15.3*	*si:ch211-217g15.3*	0.81	-0.34
miR-21	ENSDARG00000012306	*syt13*	*zgc:112063*	synaptotagmin XIII	0.80	-0.39
miR-21	ENSDARG00000014828	*l3mbtl1a*	*l3mbtl1, si:ch211-262h21.1*	L3MBTL histone methyl-lysine binding protein 1a	0.80	-0.33

miR-21	ENSDARG00000017653	*rgs13*	*zgc:163081*	regulator of G protein signaling 13	0.80	-0.33
miR-21	ENSDARG00000045352	*cst14a.2*	*cst14a, zgc:56530*	cystatin 14a, tandem duplicate 2	0.79	-0.34
miR-21	ENSDARG00000016207	*zgc:112408*	N/A	*zgc:112408*	0.79	-0.38
miR-21	ENSDARG00000062164	*Atrn*	*si:dkey-259n11.1*	attractin	0.79	-0.29
miR-21	ENSDARG00000043128	*cldne*	*cb84, fb16e12, wu:fb16e12*	claudin e	0.79	-0.32
miR-21	ENSDARG00000039434	*oprm1*	*ZFOR2, ZfMOR*	opioid receptor, mu 1	0.78	-0.29
miR-21	ENSDARG00000088505	*fam217b*	*si:dkey-211h10.3*	family with sequence similarity 217 member B	0.78	-0.37
miR-21	ENSDARG00000040910	*ildr1b*	*fc95b03, ildr1, wu:fc95b03, zgc:101133*	immunoglobulin-like domain containing receptor 1b	0.78	-0.37
miR-21	ENSDARG00000076302	*DTX4a*	*dtx1, si:ch211-119e14.2, dtx4*	deltex 4, E3 ubiquitin ligase a	0.78	-0.32
miR-21	ENSDARG00000058365	*hspb8*	*fc09c11, wu:fc04b04, wu:fc09c11, zgc:64202*	heat shock protein b8	0.78	-0.34
miR-21	ENSDARG00000026444	*apoob*	*apoo, mgc4825b, si:rp71-1f1.3, wu:fr42a11, zgc:103766*	apolipoprotein O, b	0.78	-0.30
miR-21	ENSDARG00000011459	*gsna*	*cb107, gsn, sb:cb107, u-gelsolin, wu:fi16f06*	gelsolin a	0.78	-0.24
miR-21	ENSDARG00000012381	*hsc70*	*zgc:63663*	heat shock cognate 70	0.78	-0.38
miR-21	ENSDARG00000079751	*megf8*	*fb77g03, si:dkey-108f8.4, wu:fb77g03, zgc:158741*	multiple EGF-like-domains 8	0.77	-0.29
miR-21	ENSDARG00000032010	*slc15a2*	*PEPT2, wu:fc84g07, wu:fi22a10, zgc:152684*	solute carrier family 15 member 2	0.77	-0.34
miR-21	ENSDARG00000027992	*hao2*	*zgc:63690*	hydroxyacid oxidase 2 (long chain)	0.77	-0.33
miR-21	ENSDARG00000079104	*mfhas1*	*im:7154887, si:dkey-250j7.4*	malignant fibrous histiocytoma amplified sequence 1	0.77	-0.33
miR-21	ENSDARG00000034808	*kcnip1b*	*zgc:101531*	Kv channel interacting protein 1 b	0.76	-0.33
miR-21	ENSDARG00000015863	*socs6b*	*socs6l, wu:fb77f06, zgc:64107*	suppressor of cytokine signaling 6b	0.75	-0.25
miR-21	ENSDARG00000078676	*myrf*	*c11orf9, zgc:172183*	myelin regulatory factor	0.75	-0.21
miR-21	ENSDARG00000094894	*tyrobp*	*dap12*	transmembrane immune signaling adaptor TYROBP	0.75	-0.37
miR-21	ENSDARG00000070826	*bpgm*	*zgc:92230*	2,3-bisphosphoglycerate mutase	0.75	-0.31
miR-21	ENSDARG00000060092	*zgc:165481*	N/A	*zgc:165481*	0.75	-0.36
miR-21	ENSDARG00000056572	*nrsn1l*	*si:dkey-261i16.5, NRSN2*	neurensin 1-like	0.75	-0.26
miR-21	ENSDARG00000095949	*si:dkey-22i16.9*	N/A	*si:dkey-22i16.9*	0.75	-0.30
miR-21	ENSDARG00000044924	*gdf11*	*bmp11*	growth differentiation factor 11	0.74	-0.40
miR-21	ENSDARG00000088140	*hsd17b7*	*zgc:153977*	hydroxysteroid (17-beta) dehydrogenase 7	0.74	-0.33
miR-21	ENSDARG00000009273	*ppm1da*	*ppm1d, wu:fj44c08, zgc:55836, zgc:77316*	protein phosphatase, Mg2+/Mn2+ dependent, 1Da	0.73	-0.37
miR-21	ENSDARG00000077442	*tubgcp5*	*fa03b04, fe01d03, wu:fa03b04, wu:fe01d03, zgc:110217*	tubulin, gamma complex associated protein 5	0.73	-0.31
miR-21	ENSDARG00000019335	*hes6*	*her13.2*	hes family bHLH transcription factor 6	0.73	-0.31
miR-21	ENSDARG00000046006	*med20*	*trfp, zgc:101043*	mediator complex subunit 20	0.73	-0.36
miR-21	ENSDARG00000056530	*cpamd8*	*si:ch211-87h14.4, si:dkey-94n12.1*	C3 and PZP like alpha-2-macroglobulin domain containing 8	0.73	-0.40
miR-21	ENSDARG00000073962	*ccdc32*	*si:ch211-210h14.3*	coiled-coil domain containing 32	0.72	-0.29
miR-21	ENSDARG00000024829	*tnn*	*etID309873.2, tenw, tnw, zgc:110729*	tenascin N	0.72	-0.28
miR-21	ENSDARG00000052470	*igfbp2a*	*IGFBP-2, igfbp2, igfbp2b*	insulin-like growth factor binding protein 2a	0.72	-0.21
miR-21	ENSDARG00000074866	*ptpn5*	N/A	protein tyrosine phosphatase non-receptor type 5	0.72	-0.23
miR-21	ENSDARG00000041022	*pdcd4b*	*cb45, pdcd4, sb:cb45, wu:fc84b08*	programmed cell death 4b	0.71	-0.25
miR-21	ENSDARG00000016527	*helz2*	*gb:eb955855*	helicase with zinc finger 2, transcriptional coactivator	0.71	-0.30
miR-21	ENSDARG00000053961	*slc2a11a*	*slc2a11, zglut11a, CR293534.1*	solute carrier family 2 member 11a	0.71	-0.22
miR-21	ENSDARG00000019529	*parp1*	*si:dkey-206f10.3, wu:fc60f12, zgc:110092*	poly (ADP-ribose) polymerase 1	0.71	-0.36
miR-21	ENSDARG00000037837	*ogfr*	*fb25b04, fj34c06, ogfr2, si:dkeyp-44b8.5, wu:fb25b04, wu:fj34c06*	opioid growth factor receptor	0.71	-0.25
miR-21	ENSDARG00000087867	*SLC2A13*	N/A	proton myo-inositol cotransporter-like	0.70	-0.22
miR-21	ENSDARG00000060021	*vcpkmt*	*im:7159042, mettl21d, zgc:153596*	valosin containing protein lysine (K) methyltransferase	0.70	-0.25
miR-21	ENSDARG00000031888	*mapk8a*	*jnk1a-2, zgc:172209*	mitogen-activated protein kinase 8a	0.70	-0.41
miR-21	ENSDARG00000038011	*cyth4a*	*cyth4*	cytohesin 4a	0.70	-0.26
miR-21	ENSDARG00000022659	*snx4*	*im:6971267, wu:fb92d09, zgc:113437*	sorting nexin 4	0.70	-0.32
miR-122	ENSDARG00000024443	*gnpda2*	*si:dkey-185e18.2*	glucosamine-6-phosphate deaminase 2	0.98	-0.86
miR-122	ENSDARG00000021374	*cab39l1*	*cab39, wu:fb72b01, wu:fi37c08, zgc:55451*	calcium binding protein 39, like 1	0.96	-0.60
miR-122	ENSDARG00000062954	*clk2a*	*fb20c02, si:ch211-81a5.7, wu:fb20c02, clk2*	CDC-like kinase 2a	0.96	-0.45
miR-122	ENSDARG00000031954	*rbck1*	*zgc:91964*	RanBP-type and C3HC4-type zinc finger containing 1	0.95	-0.49
miR-122	ENSDARG00000014047	*cldn7b*	*cb388, cldn7, fd19f08, wu:fd19f08*	claudin 7b	0.95	-0.43
miR-122	ENSDARG00000077364	*mapk9*	*jnk2, si:ch211-247j9.2*	mitogen-activated protein kinase 9	0.94	-0.42

miR-122	ENSDARG00000030095	*ccdc78*	N/A	coiled-coil domain containing 78	0.93	-1.18
miR-122	ENSDARG00000020326	*tyk2*	N/A	tyrosine kinase 2	0.93	-0.26
miR-122	ENSDARG00000038981	*zgc:153615*	N/A	*zgc:153615*	0.93	-0.38
miR-122	ENSDARG00000074041	*abca5*	*zgc:163009*	ATP-binding cassette, sub-family A (ABC1), member 5	0.92	-0.46
miR-122	ENSDARG00000009281	*dnm1b*	*si:ch73-310g9.3*	dynamin 1b	0.91	-0.76
miR-122	ENSDARG00000078887	*atp10d*	*si:dkey-20i10.6*	ATPase phospholipid transporting 10D	0.91	-0.59
miR-122	ENSDARG00000069507	*mettl27*	*Williams Beuren syndrome chromosome region 27, im:7139431, wbscr27, wu:fe50c11, zgc:136279*	methyltransferase like 27	0.91	-0.61
miR-122	ENSDARG00000061231	*tinagl1*	*LCN7, lipocalin-7, si:dkey-158b13.1*	tubulointerstitial nephritis antigen-like 1	0.90	-0.40
miR-122	ENSDARG00000044420	*dnajc19*	*zgc:73251*	DnaJ (Hsp40) homolog, subfamily C, member 19	0.90	-1.00
miR-122	ENSDARG00000021681	*asrgl1*	*zgc:103568*	asparaginase and isoaspartyl peptidase 1	0.90	-0.43
miR-122	ENSDARG00000060917	*Anln*	*si:ch211-197n10.2, wu:fb74d05, wu:fd10d09, wu:fi41f04*	anillin, actin binding protein	0.89	-0.41
miR-122	ENSDARG00000003776	*pip4k2aa*	*pip4k2a, zgc:194746, zgc:194777*	phosphatidylinositol-5-phosphate 4-kinase, type II, alpha a	0.89	-0.47
miR-122	ENSDARG00000075530	*Pigu*	N/A	phosphatidylinositol glycan anchor biosynthesis, class U	0.89	-0.42
miR-122	ENSDARG00000062081	*tbc1d1*	N/A	TBC1 (tre-2/USP6, BUB2, cdc16) domain family, member 1	0.89	-0.72
miR-122	ENSDARG00000068264	*grhpra*	*grhpr, si:ch211-240l14.3, zgc:109900*	glyoxylate reductase/hydroxypyruvate reductase a	0.89	-0.44
miR-122	ENSDARG00000071082	*p4ha1b*	*p4ha1, wu:fc69d06, wu:fi27h11, zgc:56058*	prolyl 4-hydroxylase, alpha polypeptide I b	0.88	-0.79
miR-122	ENSDARG00000091320	*nlrc6*	*NACHT, LRR and PYD domains-containing protein 12e, Nacht 1.01, nlrp12e, zgc:136791, zgc:136791*	NLR family CARD domain containing 6	0.88	-0.40
miR-122	ENSDARG00000017542	*fgf1a*	*fgf1, zgc:73249*	fibroblast growth factor 1a	0.88	-0.25
miR-122	ENSDARG00000061207	*ttc7b*	*wu:fj46e11, zgc:153460*	tetratricopeptide repeat domain 7B	0.87	-0.34
miR-122	ENSDARG00000000442	*slc39a13*	*ZIP13, si:by184l24.1, si:xx-184l24.1, wu:fa55a07, zgc:136440*	solute carrier family 39 member 13	0.87	-0.73
miR-122	ENSDARG00000003520	*tbc1d14*	*sb:eu1004, si:dkey-120m5.1, wu:fi35h01, wu:fi42a03*	TBC1 domain family, member 14	0.87	-0.43
miR-122	ENSDARG00000045465	*lancl2*	*zgc:158293*	LanC lantibiotic synthetase component C-like 2	0.86	-0.30
miR-122	ENSDARG00000056144	*zgc:123244*	*TMEM150B*	*zgc:123244*; orthologous to human TMEM150B (transmembrane protein 150B)	0.86	-0.47
miR-122	ENSDARG00000061436	*col6a2*	N/A	collagen, type VI, alpha 2	0.86	-0.40
miR-122	ENSDARG00000069264	*si:ch211-196h16.5*	*Gene1133*	*si:ch211-196h16.5*	0.86	-0.40
miR-122	ENSDARG00000034409	*pik3r3b*	*pik3r3, zgc:55564, zgc:85710*	phosphoinositide-3-kinase, regulatory subunit 3b (gamma)	0.85	-0.38
miR-122	ENSDARG00000038237	*tbc1d12a*	*wu:fc59a02, tbc1d12*	TBC1 domain family, member 12a	0.85	-0.52
miR-122	ENSDARG00000089444	*omgb*	*ogmb, omg*	oligodendrocyte myelin glycoprotein b	0.85	-0.44
miR-122	ENSDARG00000014181	*foxp1b*	*foxp1, im:7139058, wu:fc83a06*	forkhead box P1b	0.84	-0.30
miR-122	ENSDARG00000037837	*ogfr*	*fb25b04, fj34c06, ogfr2, si:dkeyp-44b8.5, wu:fb25b04, wu:fj34c06*	opioid growth factor receptor	0.84	-0.82
miR-122	ENSDARG00000056985	*Tpte*	*Dr-VSP, tpip, wu:fd20e11, wu:fi24b06*	transmembrane phosphatase with tensin homology	0.84	-0.40
miR-122	ENSDARG00000014674	*acsl3b*	*acsl3, fa07a08, im:7155129, si:dkey-20f20.5, wu:fa07a08, wu:fb34c03*	acyl-CoA synthetase long chain family member 3b	0.84	-0.73
miR-122	ENSDARG00000043934	*il17a/f2*	N/A	interleukin 17a/f2	0.83	-0.40
miR-122	ENSDARG00000078026	*zcchc24*	*fc45d05, wu:fc45d05, zgc:158323*	zinc finger, CCHC domain containing 24	0.83	-0.33
miR-122	ENSDARG00000076889	*ralgapa1*	*garnl1, tulip1*	Ral GTPase activating protein, alpha subunit 1 (catalytic)	0.83	-0.37
miR-122	ENSDARG00000052856	*khdrbs1a*	*fa18g12, fa56c01, fc91b01, fj90g10, khdrbs1, p62, wu:fa18g12, wu:fa56c01, wu:fc91b01, wu:fj90g10*	KH domain containing, RNA binding, signal transduction associated 1a	0.82	-0.25
miR-122	ENSDARG00000071017	*nt5e*	*zgc:63784*	5'-nucleotidase, ecto (CD73)	0.82	-0.41
miR-122	ENSDARG00000055945	*asph*	*cb971, fb69e10, fc06d04, fc95a08, junctin, wu:fb69e10, wu:fc06d04, wu:fc95a08*	aspartate beta-hydroxylase	0.82	-0.43
miR-122	ENSDARG00000073959	*pmch*	*pmch2, si:ch211-260p3.3*	pro-melanin-concentrating hormone	0.82	-0.40
miR-122	ENSDARG00000022668	*grapb*	*si:dkeyp-9d4.1, zgc:109892*	GRB2 related adaptor protein b	0.82	-0.26
miR-122	ENSDARG00000034643	*fhl3a*	*fd57e01, fhl3, wu:fd57e01, fhl3*	four and a half LIM domains 3a	0.81	-0.40
miR-122	ENSDARG00000061400	*csgalnact2*	*mp:zf637-2-001473, si:dkey-192p21.4, wu:fb98d03, zf637-2-001473*	chondroitin sulfate N-acetylgalactosaminyltransferase 2	0.80	-0.39
miR-122	ENSDARG00000055502	*cicb*	*cic, wu:fb93c01*	capicua transcriptional repressor b	0.79	-0.32
miR-122	ENSDARG00000070132	*aste1a*	*aste1, si:dkey-11p23.5*	asteroid homolog 1a	0.79	-0.48
miR-122	ENSDARG00000034240	*capza1a*	*capza1, wu:fa19e05, wu:fk22d04, zgc:56314, zgc:85654*	capping actin protein of muscle Z-line subunit alpha 1a	0.79	-0.38

miR-122	ENSDARG00000055118	*mylipb*	*zgc:153767*	myosin regulatory light chain interacting protein b	0.79	-0.42
miR-122	ENSDARG00000051814	*ptprz1a*	*ca16c, im:7144789, ptprz1, zCA16c*	protein tyrosine phosphatase receptor type Z1a	0.78	-0.40
miR-122	ENSDARG00000056762	*tm4sf21b*	*zgc:172079*	transmembrane 4 L six family member 21b	0.78	-0.45
miR-122	ENSDARG00000017244	*chd6*	*si:dkey-6e2.3, zmp:0000000556*	chromodomain helicase DNA binding protein 6	0.78	-0.25
miR-122	ENSDARG00000056184	*dvl2*	*sb:eu471, wu:fc05d12, wu:fo71e09, wu:fp54a02, zgc:55372*	dishevelled segment polarity protein 2	0.78	-0.26
miR-122	ENSDARG00000078502	*si:ch211-150o23.3*	*SSC5D*	*si:ch211-150o23.3*	0.78	-0.28
miR-122	ENSDARG00000087759	*cnih4*	*fb51e06, fc10d11, wu:fb51e06, wu:fc10d11, wu:fi84d07*	cornichon family AMPA receptor auxiliary protein 4	0.77	-0.40
miR-122	ENSDARG00000069476	*spint2*	*fb52h04, fe24a06, wu:fb52h04, wu:fe24a06, zgc:153795*	serine peptidase inhibitor, Kunitz type, 2	0.77	-0.43
miR-122	ENSDARG00000044295	*pip5k1ba*	*pip5k1b, zgc:101046*	phosphatidylinositol-4-phosphate 5-kinase, type I, beta a	0.77	-0.38
miR-122	ENSDARG00000015314	*zc4h2*	*un-named hi2729b, unm hi2729b, wu:fb99f08, zgc:63849*	zinc finger, C4H2 domain containing	0.77	-0.43
miR-122	ENSDARG00000006074	*uck2a*	*umpk, wu:fk91a01*	uridine-cytidine kinase 2a	0.77	-0.42
miR-122	ENSDARG00000062986	*pnpla7a*	*clica, zgc:158748*	patatin-like phospholipase domain containing 7a	0.77	-0.55
miR-122	ENSDARG00000056239	*tmem45b*	*zgc:77892*	transmembrane protein 45B	0.76	-0.37
miR-122	ENSDARG00000043843	*akap7*	*si:ch211-272f15.3*	A kinase (PRKA) anchor protein 7	0.76	-0.33
miR-122	ENSDARG00000033184	*prkar2aa*	*wu:fb55f12, wu:fj29e12, wu:fj33e06, zgc:153742*	protein kinase, cAMP-dependent, regulatory, type II, alpha A	0.76	-0.27
miR-122	ENSDARG00000016260	*fxr2*	*id:ibd5046, zgc:56215, zgc:77472*	fragile X mental retardation, autosomal homolog 2	0.76	-0.55
miR-122	ENSDARG00000095580	*si:ch211-67e16.11*	*wu:fk68g02*	*si:ch211-67e16.11*	0.75	-0.46
miR-122	ENSDARG00000052624	*sprydz7a*	*fd05e05, si:busm1-79f24.1, si:dkey-7a20.6, si:dz79f24.1, wu:fd05e05, zgc:56247*	SPRY domain containing 7a	0.75	-0.31
miR-122	ENSDARG00000074535	*lingo2a*	*sc:d0348, lingo2*	leucine rich repeat and Ig domain containing 2a	0.75	-0.36
miR-122	ENSDARG00000040565	*ckmb*	*ckm3, wu:fb55e09, zgc:64204*	creatine kinase, muscle b	0.75	-0.37
miR-122	ENSDARG00000086645	*hs3st3b1b*	*3-ost-3z, zf3-ost-3z*	heparan sulfate (glucosamine) 3-O-sulfotransferase 3B1b	0.75	-0.33
miR-122	ENSDARG00000038822	*mrc1b*	*mrc1, si:ch211-106h4.2*	mannose receptor, C type 1b	0.75	-0.28
miR-122	ENSDARG00000069324	*vps53*	*wu:fb77d11, zgc:103741*	VPS53 subunit of GARP complex	0.75	-0.43
miR-122	ENSDARG00000079723	*si:dkey-46g23.1*	*TNRC6B*	*si:dkey-46g23.1*; orthologous to human TNRC6B (trinucleotide repeat containing adaptor 6B)	0.75	-0.27
miR-122	ENSDARG00000077946	*smarcc2*	*baf170*	SWI/SNF related, matrix associated, actin dependent regulator of chromatin, subfamily c, member 2	0.75	-0.32
miR-122	ENSDARG00000042970	*gng8*	*zgc:110316*	guanine nucleotide binding protein (G protein), gamma 8	0.74	-0.36
miR-122	ENSDARG00000016263	*zdhhc5a*	*si:dkey-28b4.5*	zinc finger DHHC-type containing 5a	0.74	-0.30
miR-122	ENSDARG00000025309	*dpf3*	*fj43h12, si:ch211-268e23.1, wu:fj43h12*	D4, zinc and double PHD fingers, family 3	0.73	-0.43
miR-122	ENSDARG00000054848	*pdk4*	*si:rp71-57j15.4*	pyruvate dehydrogenase kinase, isozyme 4	0.73	-0.30
miR-122	ENSDARG00000056481	*vat1*	*si:dz163l24.2*	vesicle amine transport 1	0.73	-0.50
miR-122	ENSDARG00000056108	*ndufa4*	*zgc:73405*	NADH:ubiquinone oxidoreductase subunit A4	0.73	-0.42
miR-122	ENSDARG00000061473	*tbkbp1*	*zmp:0000000698*	TBK1 binding protein 1	0.72	-0.38
miR-122	ENSDARG00000077506	*tjp1a*	*cb817, tjp1, tjp1.1, zo-1*	tight junction protein 1a	0.72	-0.21
miR-122	ENSDARG00000070579	*ggact.3*	*zgc:162208, A2LD1*	gamma-glutamylamine cyclotransferase, tandem duplicate 3	0.72	-0.44
miR-122	ENSDARG00000001733	*gulp1a*	*gulp1, si:by35d8.1, si:ch211-198b21.4, si:xx-35d8.1, wuj61e02*	GULP PTB domain containing engulfment adaptor 1a	0.71	-0.25
miR-122	ENSDARG00000058114	*impad1*	*zgc:123256*	inositol monophosphatase domain containing 1	0.71	-0.29
miR-122	ENSDARG00000004939	*mtdhb*	N/A	metadherin b	0.71	-0.63
miR-122	ENSDARG00000036028	*arrdc3b*	*zgc:92034*	arrestin domain containing 3b	0.71	-0.41
miR-122	ENSDARG00000006151	*pde11al*	*pde11a, wu:ft39g12*	phosphodiesterase 11a, like	0.71	-0.21
miR-122	ENSDARG00000045360	*flj11011l*	*zgc:136503*	hypothetical protein FLJ11011-like (H. sapiens)	0.70	-0.28
miR-122	ENSDARG00000019644	*ldhba*	*ldhb, wu:fa16d07, wu:fa20c10, wu:fa96a08*	lactate dehydrogenase Ba	0.70	-0.27
miR-122	ENSDARG00000036107	*txnipa*	*sb:cb368, txnip*	thioredoxin interacting protein a	0.70	-0.34
miR-122	ENSDARG00000020890	*tmod4*	*Sk-Tmod, fa12b03, skeletal tropomodulin, sktmod, wu:fa12b03*	tropomodulin 4 (muscle)	0.70	-0.42
miR-122	ENSDARG00000037773	*rybpa*	*cb337, rybp, wu:fi34f03*	RING1 and YY1 binding protein a	0.70	-0.34
miR-731	ENSDARG00000019516	*sp7*	*osterix, osx*	Sp7 transcription factor	0.99	-0.50
miR-731	ENSDARG00000004735	*hnrnpub*	*hnrnpu, hnrpu, wu:fa66f11, wu:fc18b08, wu:fi89c08, zgc:111835, zgc:136480*	heterogeneous nuclear ribonucleoprotein Ub	0;98	-0.32
miR-731	ENSDARG00000010186	*myo3a*	N/A	myosin IIIA	0.97	-0.48

miR-731	ENSDARG00000009031	*tnikb*	*im:6898857, zgc:123033, zgc:152783*	TRAF2 and NCK interacting kinase b	0.97	-0.52
miR-731	ENSDARG00000027750	*dpp7*	*wu:fb19g02, zgc:112274, zgc:113564*	dipeptidyl-peptidase 7	0.96	-0.31
miR-731	ENSDARG00000002840	*si:dkey-28b4.8*	N/A	*si:dkey-28b4.8*	0.96	-0.26
miR-731	ENSDARG00000018923	*fat2*	*fk09f07, wu:fk09f07*	FAT atypical cadherin 2	0.96	-0.30
miR-731	ENSDARG00000032565	*cacng2a*	*cacng2, zgc:64200*	calcium channel, voltage-dependent, gamma subunit 2a	0.94	-0.30
miR-731	ENSDARG00000090764	*bcas3*	*zgc:63764*	BCAS3 microtubule associated cell migration factor	0.94	-0.61
miR-731	ENSDARG00000016854	*neurod2*	*NDF2, fj49a11, ndr2, wu:fj49a11, zNdr2*	neuronal differentiation 2	0.94	-0.30
miR-731	ENSDARG00000063572	*Perp*	*fk24g11, wu:fk24g11*	p53 apoptosis effector related to pmp22	0.93	-0.33
miR-731	ENSDARG00000062055	*rnf38*	*fa94e11, si:dkey-20n3.1, wu:fa94e11*	ring finger protein 38	0.92	-0.24
miR-731	ENSDARG00000044980	*kpna6*	*zmp:0000000623*	karyopherin alpha 6 (importin alpha 7)	0.91	-0.40
miR-731	ENSDARG00000043584	*ifit15*	*Ifit17b, ifit2, wu:fb58d07*	interferon-induced protein with tetratricopeptide repeats 15	0.90	-0.21
miR-731	ENSDARG00000070337	*hoxc1a*	*z-42*	homeobox C1a	0.90	-0.29
miR-731	ENSDARG00000019752	*rom1a*	*zgc:73336, zgc:77401*	retinal outer segment membrane protein 1a	0.90	-0.38
miR-731	ENSDARG00000024092	*lmbr1*	*im:7156444*	limb development membrane protein 1	0.90	-0.37
miR-731	ENSDARG00000057586	*sall3b*	*im:7138196, sall3l, sallb, si:dkey-195c14.1, wu:fb01e09, sall3*	spalt-like transcription factor 3b	0.90	-0.36
miR-731	ENSDARG00000042526	*sebox*	*OG9 homeobox gene, mezzo, og9x*	SEBOX homeobox	0.89	-0.38
miR-731	ENSDARG00000059707	*znf423*	*zgc:158272, si:ch211-216l23.1*	zinc finger protein 423	0.88	-0.23
miR-731	ENSDARG00000033957	*trmt44*	*mettl19, zgc:101657*	tRNA methyltransferase 44 homolog	0.88	-0.26
miR-731	ENSDARG00000068369	*angptl2b*	*angptl2, wu:fc41g02*	angiopoietin-like 2b	0.88	-0.43
miR-731	ENSDARG00000029252	*Ssb*	*wu:fb17f11*	small RNA binding exonuclease protection factor La	0.88	-0.24
miR-731	ENSDARG00000043283	*prrg1*	*zgc:103657*	proline rich Gla (G-carboxyglutamic acid) 1	0.87	-0.39
miR-731	ENSDARG00000023999	*heatr5a*	*si:busm1-142b24.3, si:dz142b24.3, zgc:63665*	HEAT repeat containing 5a	0.87	-0.43
miR-731	ENSDARG00000043003	*pcyt2*	*im:7158585, wu:fb39h11, zgc:103434*	phosphate cytidylyltransferase 2, ethanolamine	0.87	-0.53
miR-731	ENSDARG00000069922	*pla1a*	*wu:fi26h09, zgc:77160, popdc2*	popeye domain containing 2	0.87	-0.28
miR-731	ENSDARG00000019326	*prpsap2*	*wu:fi14g04, zgc:55477, zgc:85610*	phosphoribosyl pyrophosphate synthetase-associated protein 2	0.86	-0.33
miR-731	ENSDARG00000010248	*wdr54*	*zgc:100930*	WD repeat domain 54	0.86	-0.23
miR-731	ENSDARG00000016141	*slc6a2*	*si:dkey-182h7.3*	solute carrier family 6 member 2	0.86	-0.49
miR-731	ENSDARG00000062837	*spata13*	*si:dkey-33c18.3*	spermatogenesis associated 13	0.86	-0.24
miR-731	ENSDARG00000074635	*abca1a*	*abca1, cb939, fa03d03, im:6795541, wu:fa03d03, wu:fj13h01*	ATP-binding cassette, sub-family A (ABC1), member 1A	0.86	-0.27
miR-731	ENSDARG00000057869	*cdc42l2*	*cdc42b, zgc:136923*	cell division cycle 42 like 2	0.85	-0.70
miR-731	ENSDARG00000070792	*lrrc15*	*cb894, zgc:158286*	leucine rich repeat containing 15	0.85	-0.45
miR-731	ENSDARG00000054683	*prdm8b*	*si:ch211-57h10.4*	PR domain containing 8b	0.85	-0.41
miR-731	ENSDARG00000030608	*rhogd*	*rhoga, zgc:66008*	ras homolog gene family, member Gd	0.85	-0.26
miR-731	ENSDARG00000015495	*klf3*	*klf12*	Kruppel-like factor 3 (basic)	0.85	-0.26
miR-731	ENSDARG00000023323	*ywhaqb*	*wu:fa91b01, wu:fb02e12, wu:fb36f06, wu:fk86h02, ywhaq1, ywhaz, zgc:55499*	tyrosine 3-monooxygenase/tryptophan 5-monooxygenase activation protein, theta polypeptide b	0.85	-0.46
miR-731	ENSDARG00000028173	*slc4a2a*	*ae2, ae2.1, slc4a2*	solute carrier family 4 member 2a	0.84	-0.27
miR-731	ENSDARG00000051916	*cdk7*	*zgc:85821*	cyclin-dependent kinase 7	0.84	-0.28
miR-731	ENSDARG00000044492	*ublcp1*	*fb33g09, wu:fb33g09, zgc:86634*	ubiquitin-like domain containing CTD phosphatase 1	0.84	-0.24
miR-731	ENSDARG00000035837	*mosmob*	*zgc:153595, C12H16orf52*	modulator of smoothened b	0.84	-0.47
miR-731	ENSDARG00000035660	*kiaa0319l*	*si:ch211-193k19.1*	KIAA0319 like; orthologous to human *KIAA0319L*	0.83	-0.29
miR-731	ENSDARG00000040253	*onecutl*	*id:ibd5005, ocl, onecut, onecut l, onecut1*	one cut domain, family member, like	0.83	-0.62
miR-731	ENSDARG00000035634	*rfc5*	*zgc:110313*	replication factor C (activator 1) 5	0.83	-0.32
miR-731	ENSDARG00000086173	*relb*	*fj85b02, wu:fj85b02*	bv-rel avian reticuloendotheliosis viral oncogene homolog B	0.83	-0.29
miR-731	ENSDARG00000004160	*reep3b*	*im:6909180, reep3, sb:eu299, zgc:55529*	receptor accessory protein 3b	0.83	-0.24
miR-731	ENSDARG00000056292	*vsx1*	*etID309864.17*	visual system homeobox 1 homolog, chx10-like	0.83	-0.53
miR-731	ENSDARG00000077721	*knop1*	*si:ch211-202h22.6, C1H16orf88*	lysine-rich nucleolar protein 1	0.83	-0.23
miR-731	ENSDARG00000043019	*exoc1*	*sec3l1, wu:fb58e03, zgc:64145*	exocyst complex component 1	0.82	-0.24
miR-731	ENSDARG00000056347	*rab3aa*	*rab3a, zgc:92276*	RAB3A, member RAS oncogene family, a	0.82	-0.27
miR-731	ENSDARG00000016743	*angel2*	*fl78e02, si:ch211-181h6.2, wu:fl78e02, zgc:153829*	angel homolog 2 (Drosophila)	0.82	-0.27
miR-731	ENSDARG00000032496	*pon1*	*zgc:91887*	paraoxonase 1	0.82	-0.25
miR-731	ENSDARG00000008788	*camk1gb*	*fk61c03, wu:fk61c03, zgc:73155*	calcium/calmodulin-dependent protein kinase IGb	0.82	-0.29

miR-731	ENSDARG00000010454	*guca1a*	*gcap1*	guanylate cyclase activator 1A	0.81	-0.35
miR-731	ENSDARG00000001676	*gpm6bb*	*DMgamma2, gpb6bb, zgc:110793*	glycoprotein M6Bb	0.81	-0.41
miR-731	ENSDARG00000059575	*uhmk1*	*zgc:153241*	U2AF homology motif (UHM) kinase 1	0.81	-0.28
miR-731	ENSDARG00000079740	*efna2b*	*si:dkey-174e10.1, EFNA2*	ephrin-A2b	0.81	-0.25
miR-731	ENSDARG00000013730	*slc4a4a*	*NBC1, NBCe1, id:ibd2520, si:dkey-256m11.1, slc4a4*	solute carrier family 4 member 4a	0.81	-0.59
miR-731	ENSDARG00000010791	*dla*	*cb335, delta A, fa04c10, wu:fa04c10*	deltaA	0.80	-0.26
miR-731	ENSDARG00000022372	*kng1*	*fb64g01, wu:fb64g01, zgc:103569*	kininogen 1	0.80	-0.27
miR-731	ENSDARG00000079345	*si:ch211-217k17.10*	*fk24c04, wu:fk24c04*	*si:ch211-217k17.10*	0.80	-0.57
miR-731	ENSDARG00000030871	*siah1*	*id:ibd2635, zgc:56026*	siah E3 ubiquitin protein ligase 1	0.80	-0.37
miR-731	ENSDARG00000043334	*ccdc6a*	*ccdc6, fj36e04, wu:fj36e04, zgc:77435*	coiled-coil domain containing 6a	0.80	-0.59
miR-731	ENSDARG00000056150	*rbms2b*	*zgc:100836*	RNA binding motif, single stranded interacting protein 2b	0.80	-0.22
miR-731	ENSDARG00000020607	*emc3*	*loc55831, pob, wu:fi32f05, wu:fj63h08, zgc:63727*	ER membrane protein complex subunit 3	0.79	-0.76
miR-731	ENSDARG00000070955	*hmx3a*	*Nkx5-1, hmx3, nkx5.1, zgc:109845*	H6 family homeobox 3a	0.79	-0.22
miR-731	ENSDARG00000035577	*cds2*	*zgc:66134*	CDP-diacylglycerol synthase (phosphatidate cytidylyltransferase) 2	0.79	-0.27
miR-731	ENSDARG00000076710	*si:dkey-42l23.7*	*zmp:0000000806, CR848836.6*	*si:dkey-42l23.7;* orthologous to human *GPR33* (G protein-coupled receptor 33)	0.79	-0.21
miR-731	ENSDARG00000009273	*ppm1da*	*ppm1d, wu:fj44c08, zgc:55836, zgc:77316*	protein phosphatase, Mg2+/Mn2+ dependent, 1Da	0.78	-0.50
miR-731	ENSDARG00000043608	*eif4ebp1*	*fc04h09, wu:fc04h09, zgc:64137*	eukaryotic translation initiation factor 4E binding protein 1	0.78	-0.23
miR-731	ENSDARG00000091433	*abhd17aa*	*abhd17ca, fb50g01, wu:fb50g01, zgc:162293, FAM108A1*	abhydrolase domain containing 17Aa	0.78	-0.24
miR-731	ENSDARG00000044267	*sumo1*	*fd12c02, hm:zeh0670, wu:fb74c02, wu:fd12c02, zgc:65934, zgc:85634*	small ubiquitin like modifier 1	0.78	-0.52
miR-731	ENSDARG00000094377	*samd10b*	*samd10, si:ch211-278f9.1, samd10*	sterile alpha motif domain containing 10b	0.78	-0.24
miR-731	ENSDARG00000042961	*zgc:100920*	N/A	*zgc:100920*; orthologous to human *NKAIN4* (sodium/potassium transporting ATPase interacting 4).	0.78	-0.40
miR-731	ENSDARG00000053110	*pkib*	*im:7054531, si:dkey-150h13.1*	protein kinase (cAMP-dependent, catalytic) inhibitor beta	0.78	-0.27
miR-731	ENSDARG00000017740	*sec63*	*zgc:92718*	SEC63 homolog, protein translocation regulator	0.78	-0.39
miR-731	ENSDARG00000078095	*cipcb*	*si:ch73-387e10.3*	CLOCK-interacting pacemaker b	0.78	-0.22
miR-731	ENSDARG00000011611	*actr1*	*hm:zehn1110, wu:fc22g09, wu:fc56d01, wu:fy52e08, zehn1110, zgc:56317*	actin related protein 1, centractin	0.78	-0.21
miR-731	ENSDARG00000053665	*gabrg2*	*si:ch211-145n14.1*	gamma-aminobutyric acid (GABA) A receptor, gamma 2	0.78	-0.29
miR-731	ENSDARG00000058256	*draxin*	*neucrin, zgc:113312, C11H1orf187*	dorsal inhibitory axon guidance protein	0.78	-0.26
miR-731	ENSDARG00000070918	*si:ch211-284e20.8*	*FETUB*	*si:ch211-284e20.8*; orthologous to human *FETUB* (fetuin B)	0.77	-0.22
miR-731	ENSDARG00000020031	*cldn11a*	*zgc:92247*	claudin 11a	0.77	-0.22
miR-731	ENSDARG00000018621	*slc6a19a.1*	*slc6a19a, zgc:162095*	solute carrier family 6 member 19a, tandem duplicate 1	0.77	-0.25
miR-731	ENSDARG00000015860	*mtmr9*	*si:dkey-155d18.2*	myotubularin related protein 9	0.77	-0.26
miR-731	ENSDARG00000058082	*birc7*	*zgc:165605*	baculoviral IAP repeat containing 7	0.77	-0.54
miR-731	ENSDARG00000092501	*si:dkey-80c24.5*	N/A	*si:dkey-80c24.5*	0.77	-0.32
miR-731	ENSDARG00000012248	*rgma*	*id:ibd2030, wu:fb72h09, zgc:110534*	repulsive guidance molecule BMP co-receptor a	0.77	-0.49
miR-731	ENSDARG00000017886	*ZBTB11*	*man, marsanne, mne, si:dkey-98p3.1, si:dkeyp-116g9.2, wu:fc06h02, wu:fe36e08*	zinc finger and BTB domain containing 11	0.77	-0.44
miR-731	ENSDARG00000029290	*stx11b.1*	*zgc:77855*	syntaxin 11b, tandem duplicate 1	0.76	-0.25
miR-731	ENSDARG00000020134	*sipa1l1*	*cb600, id:ibd5092, sb:cb600, si:ch211-106p8.1, wu:fc43g06*	signal-induced proliferation-associated 1 like 1	0.76	-0.21
miR-731	ENSDARG00000058535	*si:dkey-211g8.7*	*FFAR2*	*si:dkey-211g8.7*; orthologous to human *FFAR2* (free fatty acid receptor 2)	0.76	-0.26
miR-731	ENSDARG00000061738	*march2*	*si:ch211-197g15.3, zgc:158704*	membrane-associated ring finger (C3HC4) 2	0.76	-0.22
miR-731	ENSDARG00000074363	*TTC9*	N/A	*si:ch211-259k16.3*	0.76	-0.31
miR-731	ENSDARG00000021539	*ephb2b*	*fi18g12, wu:fi18g12, zgc:112296*	eph receptor B2b	0.76	-0.25
miR-731	ENSDARG00000028379	*trub2*	*fc51e05, wu:fc51e05, zgc:91836*	TruB pseudouridine (psi) synthase family member 2	0.76	-0.26
miR-731	ENSDARG00000004954	*grna*	*grn, pgrn-a, progranulin, sb:eu337, wu:fb11g12, wu:fj18f05, zfPGRN-A*	granulin a	0.76	-0.28
miR-731	ENSDARG00000039008	*SERP2*	*fc56c08, wu:fc56c08, zgc:85858*	*zgc:85858*	0.75	-0.24
miR-731	ENSDARG00000008904	*smarca2*	*wu:fa56c07, wu:fi27f11, zgc:66238*	SWI/SNF related, matrix associated, actin dependent regulator of chromatin, subfamily a, member 2	0.75	-0.32

miR-731	ENSDARG00000090889	*si:ch211-132p1.3*	*zmp:0000001007, BX005069.4, lect2*	*si:ch211-132p1.3*; orthologous to human *LECT2* (leukocyte cell derived chemotaxin 2)	0.75	-0.25
miR-731	ENSDARG00000063544	*pip4k2ab*	*si:ch211-279l9.5*	phosphatidylinositol-5-phosphate 4-kinase, type II, alpha b	0.75	-0.33
miR-731	ENSDARG00000078492	*swi5*	*si:ch211-264f7.1*	SWI5 homologous recombination repair protein	0.75	-0.37
miR-731	ENSDARG00000074809	*zgc:194392*	*fd59g01, wu:fd59g01*	*zgc:194392*; orthologous to human *C1orf115* (chromosome 1 open reading frame 115)	0.75	-0.26
miR-731	ENSDARG00000031809	*rbm24b*	*zgc:136803*	RNA binding motif protein 24b	0.75	-0.36
miR-731	ENSDARG00000037177	*zc3h13*	*zgc:66359*	zinc finger CCCH-type containing 13	0.74	-0.26
miR-731	ENSDARG00000059474	*mlxip*	*Mlx, chrebp, si:ch211-110p13.4, wu:fb38e04*	MLX interacting protein	0.74	-0.37
miR-731	ENSDARG00000011885	*mrpl19*	*wu:ft69e01, zgc:100827*	mitochondrial ribosomal protein L19	0.74	-0.42
miR-731	ENSDARG00000027183	*namptb*	*im:7051384, wu:fc21g12, wu:fc27h03, wu:fd15d03, zgc:55764*	nicotinamide phosphoribosyltransferase b	0.74	-0.22
miR-731	ENSDARG00000074262	*nck1a*	*si:dkey-4m11.4, nck1*	NCK adaptor protein 1a	0.74	-0.23
miR-731	ENSDARG00000038737	*phf20b*	*zgc:91986*	PHD finger protein 20, b	0.74	-0.25
miR-731	ENSDARG00000094491	*si:ch211-202m22.1*	N/A	*si:ch211-202m22.1*	0.74	-0.31
miR-731	ENSDARG00000074367	*usp12b*	*CR381676.2*	ubiquitin specific peptidase 12b	0.74	-0.25
miR-731	ENSDARG00000007125	*asb6*	*zgc:110231*	ankyrin repeat and SOCS box containing 6	0.74	-0.36
miR-731	ENSDARG00000018643	*igf2a*	*IGF-II, etID31232.25, igf2, igf2b, wu:fc26e03, zgc:91781*	insulin-like growth factor 2a	0.74	-0.31
miR-731	ENSDARG00000028552	*plppr3b*	*PRG2A, lppr3, lppr3b, BX000430.1*	phospholipid phosphatase related 3b	0.74	-0.32
miR-731	ENSDARG00000074597	*lzts2a*	*lzts2, si:dkey-250a11.1*	leucine zipper, putative tumor suppressor 2a	0.74	-0.39
miR-731	ENSDARG00000054344	*gabpb2b*	*im:7142141, si:dkey-77n11.3, zgc:154003*	GA binding protein transcription factor subunit beta 2b	0.73	-0.40
miR-731	ENSDARG00000073813	*scly*	*zgc:171514*	selenocysteine lyase	0.73	-0.40
miR-731	ENSDARG00000079784	*si:ch211-235o23.1*	*TMEM71*	*si:ch211-235o23.1*; orthologous to human *TMEM71* (transmembrane protein 71)	0.73	-0.29
miR-731	ENSDARG00000008979	*golga1*	*zgc:63688*	golgin A1	0.73	-0.25
miR-731	ENSDARG00000035398	*enc1*	*klhl37, si:dkey-35i22.1*	ectodermal-neural cortex 1	0.73	-0.22
miR-731	ENSDARG00000055305	*ret*	*c-ret, cret, etID315074.13, ret1, wu:fd13h01*	ret proto-oncogene receptor tyrosine kinase	0.72	-0.36
miR-731	ENSDARG00000043640	*cenpn*	*id:ibd2033, si:busm1-241h12.3, si:dz241h12.3, un-named hi3634, unm hi3634, zgc:92188*	centromere protein N	0.72	-0.28
miR-731	ENSDARG00000059244	*arfip2a*	*fj35g06, wu:fj35g06, zgc:92579*	ADP-ribosylation factor interacting protein 2a	0.72	-0.24
miR-731	ENSDARG00000061992	*dot1l*	*si:dkey-211f22.1*	DOT1-like histone H3K79 methyltransferase	0.72	-0.21
miR-731	ENSDARG00000069833	*rhbdl2*	*zgc:55533*	rhomboid, veinlet-like 2 (Drosophila)	0.72	-0.31
miR-731	ENSDARG00000011553	*atp5pb*	*atp5f1, fb59g11, fj08b08, wu:fb59g11, wu:fj08b08, zgc:101887*	ATP synthase peripheral stalk-membrane subunit b	0.71	-0.42
miR-731	ENSDARG00000006010	*bmi1a*	*bmi1, pcgf4, psc1, wu:fb17g03, wu:fd18f06*	bmi1 polycomb ring finger oncogene 1a	0.71	-0.22
miR-731	ENSDARG00000077265	*wdr89*	N/A	WD repeat domain 89	0.71	-0.23
miR-731	ENSDARG00000007216	*abce1*	*wu:fb34c09, wu:fe47b01, wu:fi09g07, zgc:111906, zgc:56045*	ATP-binding cassette, sub-family E (OABP), member 1	0.71	-0.34
miR-731	ENSDARG00000070567	*cadpsb*	N/A	Ca^{2+}-dependent activator protein for secretion b	0.71	-0.31
miR-731	ENSDARG00000044827	*wnt7aa*	*wnt7a*	wingless-type MMTV integration site family, member 7Aa	0.71	-0.54
miR-731	ENSDARG00000076170	*pcsk1nl*	*proSAAS, uo:ion003, wu:fj32g06*	proprotein convertase subtilisin/kexin type 1 inhibitor, like	0.70	-0.31
miR-731	ENSDARG00000077096	*fndc7a*	*fndc7, si:dkeyp-51f11.6*	fibronectin type III domain containing 7a	0.70	-0.24
miR-731	ENSDARG00000079414	*sez6b*	N/A	seizure related 6 homolog b	0.70	-0.21
miR-731	ENSDARG00000078576	*setd5*	N/A	SET domain containing 5	0.70	-0.38
miR-731	ENSDARG00000091817	*CR391998.1*	N/A	putative protein TPRXL	0.70	-0.29
miR-731	ENSDARG00000053857	*ccdc187*	*zgc:162431*	coiled-coil domain containing 187	0.70	-0.24
miR-731	ENSDARG00000042115	*tmem198a*	*si:rp71-68g1.6*	transmembrane protein 198a	0.70	-0.31
miR-731	ENSDARG00000020278	*si:dkeyp-120h9.1*	*N/A*	*si:dkeyp-120h9.1*; orthologous to human *SLC7A13* (solute carrier family 7 member 13)	0.70	-0.72
miR-26	ENSDARG00000059801	*pdzd2*	*si:dkey-3h3.1*	PDZ domain containing 2	0.96/0.97*	-0.58
miR-26	ENSDARG00000087176	*rskra*	*rskr, sgk494a, si:dkeyp-118e10.5, AL954372.2*	ribosomal protein S6 kinase related a	0.96/0.96	-0.51
miR-26	ENSDARG00000025766	*zgc:66440*	*fi25g12, wu:fi25g12, DTX3*	*zgc:66440*	0.72/-	-0.48
miR-26	ENSDARG00000055108	*gde1*	*zgc:56068, zgc:77135*	glycerophosphodiester phosphodiesterase 1	0.94/0.94	-0.47
miR-26	ENSDARG00000009901	*slc38a5a*	*fk81c02, si:dkey-48j7.5, wu:fk81c02*	solute carrier family 38 member 5a	0.93/0.91	-0.46

miR-26	ENSDARG00000040684	*plcd4b*	*DrPLC-delta 4B, plcd4*	phospholipase C, delta 4b	0.83/0.84	-0.46
miR-26	ENSDARG00000074301	*cth*	*fb58d08, wu:fb48e03, wu:fb58d08, zgc:66001, zgc:85785*	cystathionase (cystathionine gamma-lyase)	0.91/0.90	-0.44
miR-26	ENSDARG00000052438	*actr2a*	*actr2, hm:zeh1257, zgc:63719*	actin related protein 2a	0.78/0.80	-0.43
miR-26	ENSDARG00000078585	*mon1a*	*zgc:162873*	MON1 secretory trafficking family member A	0.91/0.90	-0.43
miR-26	ENSDARG00000044183	*prkab1a*	*zgc:92228*	protein kinase, AMP-activated, beta 1 non-catalytic subunit, a	0.73/0.71	-0.43
miR-26	ENSDARG00000001898	*manea*	*fi29h09, wu:fi29h09, zgc:92825*	mannosidase, endo-alpha	0.90/0.91	-0.42
miR-26	ENSDARG00000043026	*ttbk2b*	*fj62e10, sb:eu1102, si:dkey-12h9.11, si:dkey-12h9.14, wu:fj62e10*	tau tubulin kinase 2b	0.81/0.81	-0.41
miR-26	ENSDARG00000028027	*trim63a*	*MuRF1A, fc50c07, murf1, trim63, wu:fc50c07, zgc:86757*	tripartite motif containing 63a	0.91/0.93	-0.40
miR-26	ENSDARG00000036066	*si:dkey-78a14.4*	N/A	*si:dkey-78a14.4*; orthologous to several human genes including *NAT1* (N-acetyltransferase 1)	0.70/-	-0.40
miR-26	ENSDARG00000006220	*ugt1a1*	*ugt1ab*	UDP glucuronosyltransferase 1 family, polypeptide A1	0.93/0.92	-0.39
miR-26	ENSDARG00000052386	*cldnd1b*	*cldnd1*	claudin domain containing 1b	0.82/0.81	-0.38
miR-26	ENSDARG00000017427	*phf20l1*	*fi20g04, si:dkey-97i18.2, wu:fi20g04*	PHD finger protein 20 like 1	0.95/0.95	-0.38
miR-26	ENSDARG00000003259	*loxa*	*lox, zgc:77447*	lysyl oxidase a	0.94/0.94	-0.37
miR-26	ENSDARG00000039966	*prom1a*	*fb75c01, prom1, proml1, wu:fb75c01*	prominin 1a	0.88/0.87	-0.37
miR-26	ENSDARG00000015184	*mpp3a*	*mpp3, si:ch73-368i2.1, zmp:0000000552*	membrane protein, palmitoylated 3a (MAGUK p55 subfamily member 3)	0.86/0.86	-0.37
miR-26	ENSDARG00000088377	*rp1l1b*	*si:dkey-33i22.3*	*rp1 like 1b*; orthologous to human *RP1L1*	0.88/0.88	-0.36
miR-26	ENSDARG00000002696	*gnb3b*	*gnb3, wu:fk54b04, zgc:73058, zgc:77780*	guanine nucleotide binding protein (G protein), beta polypeptide 3b	0.86/0.86	-0.36
miR-26	ENSDARG00000038296	*tmem86b*	*zgc:153124*	transmembrane protein 86B	0.82/0.81	-0.36
miR-26	ENSDARG00000091548	*stard9*	*BX569781.2*	StAR-related lipid transfer (START) domain containing 9	0.91/0.90	-0.36
miR-26	ENSDARG00000045586	*zgc:172145*	N/A	*zgc:172145*; orthologous to human *FTL* (ferritin light chain)	0.96/0.97	-0.36
miR-26	ENSDARG00000058486	*caps2*	*si:ch73-86n2.3*	calcyphosine 2	0.78/0.78	-0.36
miR-26	ENSDARG00000007485	*epc2*	*zgc:55784*	enhancer of polycomb homolog 2 (Drosophila)	0.99/0.99	-0.35
miR-26	ENSDARG00000028088	*galk1*	*zgc:101541*	galactokinase 1	0.79/0.81	-0.35
miR-26	ENSDARG00000016584	*rgs7a*	*rgs7, zgc:92793*	regulator of G protein signaling 7a	0.83/0.85	-0.35
miR-26	ENSDARG00000016141	*slc6a2*	*si:dkey-182h7.3*	solute carrier family 6 member 2	0.76/0.76	-0.35
miR-26	ENSDARG00000021255	*arhgap22*	*si:ch211-223a10.2*	Rho GTPase activating protein 22	-/0.71	-0.35
miR-26	ENSDARG00000076844	*plin6*	*zgc:162150*	perilipin 6	0.72/0.72	-0.34
miR-26	ENSDARG00000005236	*srcap*	*im:7137889, im:7138264*	Snf2-related CREBBP activator protein	0.90/0.90	-0.34
miR-26	ENSDARG00000058978	*zgc:113423*	*BEND2*	*zgc:113423*; orthologous to human *BEND2* (BEN domain containing 2)	0.98/0.96	-0.34
miR-26	ENSDARG00000069045	*chtf8*	*si:ch211-218c6.5, zgc:158641*	CTF8, chromosome transmission fidelity factor 8 homolog (S. cerevisiae)	0.78/0.79	-0.34
miR-26	ENSDARG00000063414	*rxylt1*	*tmem5, zgc:153239*	ribitol xylosyltransferase 1	0.91/0.93	-0.34
miR-26	ENSDARG00000079500	*kif3cb*	*kif3c, kif3c-like*	kinesin family member 3Cb	0.80/0.79	-0.34
miR-26	ENSDARG00000022550	*gxylt1b*	*glt8d3, gxylt1, si:ch211-155a11.6*	glucoside xylosyltransferase 1b	0.91/0.90	-0.34
miR-26	ENSDARG00000016022	*zic5*	N/A	zic family member 5 (odd-paired homolog, Drosophila)	0.99/0.99	-0.34
miR-26	ENSDARG00000060354	*samd7*	*si:dkey-52k20.12*	sterile alpha motif domain containing 7	0.87/0.87	-0.33
miR-26	ENSDARG00000060921	*pla2g6*	*wu:fc06g09, zgc:77476*	phospholipase A2, group VI (cytosolic, calcium-independent)	0.84/0.82	-0.33
miR-26	ENSDARG00000003167	*mlst8*	*fi37e04, gbl, wu:fi37e04, zgc:55455, zgc:85668*	MTOR associated protein, LST8 homolog (S. cerevisiae)	0.94/0.94	-0.33
miR-26	ENSDARG00000075612	*ercc6*	N/A	excision repair cross-complementation group 6	0.85/0.86	-0.33
miR-26	ENSDARG00000044513	*tpst1l*	N/A	tyrosylprotein sulfotransferase 1, like	0.77/0.75	-0.33
miR-26	ENSDARG00000058608	*wsb2*	*si:ch73-263f13.4*	WD repeat and SOCS box containing 2	0.79/0.82	-0.33
miR-26	ENSDARG00000052121	*rag2*	*zgc:136743*	recombination activating gene 2	0.82/0.83	-0.33
miR-26	ENSDARG00000002771	*slc4a5a*	*si:ch211-48m9.1*	*solute carrier family 4 member 5a*	0.89/0.88	-0.33
miR-26	ENSDARG00000058548	*bves*	*pop1, popdc1, zgc:86887*	blood vessel epicardial substance	0.76/0.76	-0.33
miR-26	ENSDARG00000039563	*loxl3b*	*fj60c12, im:7157119, wu:fj60c12, zgc:162959*	lysyl oxidase-like 3b	0.75/0.74	-0.32
miR-26	ENSDARG00000045601	*cax1*	*si:dkey-180p18.2, wu:fc25f10, zgc:136271*	cation/H+ exchanger protein 1	0.81/0.80	-0.32
miR-26	ENSDARG00000044325	*zgc:193690*	*sb:eu534, zgc:193696*	*zgc:193690*; Orthologous to human iand *DDX19B* (DEAD-box helicase 19A and 19B)	0.83/0.83	-0.32
miR-26	ENSDARG00000069538	*gtf3c4*	*im:7138272, si:dkeyp-10e10.3*	general transcription factor IIIC, polypeptide 4	0.76/0.75	-0.32
miR-26	ENSDARG00000034427	*jpt2*	*fc04g11, hn1l, wu:fb37f03, wu:fc04g11, zgc:55712*	Jupiter microtubule associated homolog 2	0.77/0.77	-0.32
miR-26	ENSDARG00000079712	*gal3st4*	*fb92g05, wu:fb92g05*	galactose-3-O-sulfotransferase 4	0.83/0.84	-0.32
miR-26	ENSDARG00000058357	*ankrd33ba*	*si:rp71-1f1.8*	ankyrin repeat domain 33ba	0.71/0.72	-0.32

miR-26	ENSDARG00000045911	*tulp4a*	*fb99g11, si:ch211-222h17.4, si:ch211-51e12.5, tulp4, wu:fb75h04, wu:fb99g11*	TUB like protein 4a	0.87/0.89	-0.31
miR-26	ENSDARG00000021664	*fzd3a*	*fz3a, fz9, fzd3, off-limits, olt, zg09*	frizzled class receptor 3a	0.90/0.89	-0.31
miR-26	ENSDARG00000041081	*kmt5b*	*suv420h1, wu:fb97g06, wu:fi57g03, zgc:103527*	lysine methyltransferase 5B	0.88/0.88	-0.31
miR-26	ENSDARG00000039390	*sdhaf4*	*si:busm1-265n4.4, si:busm1-46p4.2, si:ch211-103o12.3, C13H6orf57*	succinate dehydrogenase complex assembly factor 4	0.85/0.82	-0.31
miR-26	ENSDARG00000069540	*si:dkey-30c15.2*	N/A	*si:dkey-30c15.2*	0.85/0.87	-0.31
miR-26	ENSDARG00000054937	*badb*	*bad, fa01b12, proapoptotic BH3-only protein, wu:fa01b12, wu:fa96d04*	BCL2 associated agonist of cell death b	0.93/0.93	-0.31
miR-26	ENSDARG00000079730	*fuz*	N/A	fuzzy planar cell polarity protein	0.92/0.90	-0.31
miR-26	ENSDARG00000009021	*chrna1*	*AChR alpha 1, nic, nic-1, nic1, nicotinic receptor, zgc:86593*	cholinergic receptor, nicotinic, alpha 1 (muscle)	0.76/0.76	-0.31
miR-26	ENSDARG00000030512	*tsnaxip1*	*wu:fc26b06, zgc:162756*	translin-associated factor X interacting protein 1	0.89/0.89	-0.31
miR-26	ENSDARG00000017741	*g3bp1*	*fj17h05, wu:fj17h05, zgc:56034*	GTPase activating protein (SH3 domain) binding protein 1	0.94/0.94	-0.31
miR-26	ENSDARG00000078592	*nomo*	*loc283820*	nodal modulator	0.80/0.74	-0.31
miR-26	ENSDARG00000088290	*foxj1b*	*fj67h07, foxj1.2, wu:fj67h07, zgc:101713*	forkhead box J1b	0.74/0.75	-0.31
miR-26	ENSDARG00000074527	*chst15*	*si:dkey-59k9.3*	carbohydrate (N-acetylgalactosamine 4-sulfate 6-O) sulfotransferase 15	0.86/0.82	-0.30
miR-26	ENSDARG00000077950	*tor1*	*im:7165310, si:ch73-178d14.1*	torsin family 1	0.73/0.73	-0.30
miR-26	ENSDARG00000022183	*gsto1*	*fd58h04, wu:fd58h04, zgc:92254*	glutathione S-transferase omega 1	0.81/0.84	-0.30
miR-26	ENSDARG00000011605	*dennd6b*	*fam116b, fj67e07, wu:fj67e07, zgc:55619*	DENN/MADD domain containing 6B	0.90/0.88	-0.30
miR-26	ENSDARG00000027199	*smad1*	*SMA- and MAD-related protein 1, fb39h09, madh1, sb:eu786, wu:fb39h09, wu:fb97e04*	SMAD family member 1	0.93/0.92	-0.30
miR-26	ENSDARG00000055070	*borcs7*	*zgc:114169, C13H10orf32*	BLOC-1 related complex subunit 7	0.70/0.70	-0.30
miR-26	ENSDARG00000092949	*si:ch73-366i20.1*	N/A	*si:ch73-366i20.1*	0.76/0.79	-0.30
miR-26	ENSDARG00000068551	*elovl8a*	*zgc:153394*	ELOVL fatty acid elongase 8a	0.76/0.76	-0.30
miR-26	ENSDARG00000020618	*gatd3a*	*zgc:112056, C9H21orf33*	glutamine amidotransferase like class 1 domain containing 3A	0.77/0.77	-0.30
miR-26	ENSDARG00000030616	*nfe2l1a*	*nfe2l1, nrf1a*	nuclear factor, erythroid 2-like 1a	0.88/0.88	-0.30
miR-26	ENSDARG00000060383	*mmaa*	*zgc:171657*	metabolism of cobalamin associated A	0.88/0.87	-0.29
miR-26	ENSDARG00000002959	*gpr34b*	*zgc:110315*	G protein-coupled receptor 34b	0.84/0.83	-0.29
miR-26	ENSDARG00000012025	*hs6st2*	*fb80f12, hs6st, wu:fb80f12*	heparan sulfate 6-O-sulfotransferase 2	0.84/0.82	-0.29
miR-26	ENSDARG00000073948	*prok1*	*zgc:171925*	prokineticin 1	0.70/0.70	-0.29
miR-26	ENSDARG00000040607	*rtf2*	*rtfdc1, wu:fd99e08, zgc:55448, zgc:86650, C6H20orf43*	replication termination factor 2	0.90/0.91	-0.29
miR-26	ENSDARG00000075721	*zdhhc6*	*fa04d12, fc30b09, wu:fa04d12, wu:fc30b09*	zinc finger DHHC-type containing 6	0.90/0.90	-0.29
miR-26	ENSDARG00000070256	*suz12a*	*suz12, suz12l, zgc:100863*	SUZ12 polycomb repressive complex 2 subunit a	0.71/0.71	-0.29
miR-26	ENSDARG00000036864	*slc34a2b*	*NaPi-IIb2, wu:fi68d06*	solute carrier family 34 member 2b	0.76/0.75	-0.29
miR-26	ENSDARG00000012860	*tmprss4b*	*si:ch211-139a5.13, si:ch211-139a5.6, wu:fi21e09, wu:fk84e06*	transmembrane serine protease 4b	0.90/0.87	-0.29
miR-26	ENSDARG00000078891	*map11*	*C7orf43*	microtubule associated protein 1	0.81/0.81	-0.29
miR-26	ENSDARG00000012932	*mat2b*	*fc55d01, wu:fb48h02, wu:fc55d01, zgc:110308*	methionine adenosyltransferase II, beta	0.76/0.76	-0.29
miR-26	ENSDARG00000032103	*mapk6*	*ERK3, si:ch211-235f12.1*	mitogen-activated protein kinase 6	0.84/0.81	-0.29
miR-26	ENSDARG00000017803	*gsk3ba*	*GSK-3[b], fb68h05, fk80d11, gsk3b, wu:fb68h05, wu:fk80d11*	glycogen synthase kinase 3 beta, genome duplicate a	0.84/0.85	-0.28
miR-26	ENSDARG00000035986	*ptpn2b*	*cb806, ptpn2, ptpn2l, wu:fc10h07, zgc:55339, zgc:76973*	protein tyrosine phosphatase non-receptor type 2b	0.85/0.85	-0.28
miR-26	ENSDARG00000043857	*ufm1*	*ubfm1, wu:fa10f05, wu:fb72g01, zgc:55335*	ubiquitin-fold modifier 1	0.72/0.71	-0.28
miR-26	ENSDARG00000010841	*fshb*	*FSH Beta, FSHB, Gth I, gthI*	follicle stimulating hormone subunit beta	0.88/0.88	-0.28
miR-26	ENSDARG00000054208	*phkg2*	*zgc:55863*	phosphorylase kinase, gamma 2 (testis)	0.86/0.86	-0.28
miR-26	ENSDARG00000040161	*zgc:92287*	N/A	*zgc:92287*	0.75/0.74	-0.28
miR-26	ENSDARG00000056196	*slc2a2*	*wu:fb62h10, zglut2*	solute carrier family 2 member 2	0.77/0.77	-0.28
miR-26	ENSDARG00000009681	*med27*	*crsp34, crsp8, trap37, zgc:66302*	mediator complex subunit 27	0.83/0.83	-0.28
miR-26	ENSDARG00000024503	*c6ast3*	*zgc:103606*	six-cysteine containing astacin protease 3	0.75/0.75	-0.28
miR-26	ENSDARG00000091678	*ints12*	*wu:fi17f05, zgc:86602*	integrator complex subunit 12	0.78/0.77	-0.28
miR-26	ENSDARG00000008986	*e2f7*	*fc43h03, si:dkey-217k21.2, wu:fc43h03*	E2F transcription factor 7	0.77/0.76	-0.28
miR-26	ENSDARG00000053569	*sox3*	*id:ibd2036, sb:cb493, wu:fd02a08, zgc:110279*	SRY-box transcription factor 3	0.92/0.92	-0.28
miR-26	ENSDARG00000093378	*si:ch211-235i11.5*	*CR382385.3*	*si:ch211-235i11.5*	0.82/0.82	-0.28

miR-26	ENSDARG00000018190	*get3*	*asna1, trc40, wu:fd05d03, zgc:56540, zgc:86799*	guided entry of tail-anchored proteins factor 3, ATPase	0.80/0.80	-0.28
miR-26	ENSDARG00000016837	*glipr2l*	*wu:fj53e10, zgc:103578*	GLI pathogenesis-related 2, like	0.80/0.80	-0.28
miR-26	ENSDARG00000018643	*igf2a*	*IGF-II, etID31232.25, igf2, igf2b, wu:fc26e03, zgc:91781*	insulin-like growth factor 2a	0.79/0.79	-0.28
miR-26	ENSDARG00000025679	*comtb*	*si:dkey-13a21.15, zgc:162236*	catechol-O-methyltransferase b	0.75/0.75	-0.28
miR-26	ENSDARG00000035719	*arl5c*	*zgc:77751*	ADP-ribosylation factor-like 5C	0.77/0.76	-0.28
miR-26	ENSDARG00000052527	*mul1b*	*im:7146383, zgc:165594*	mitochondrial E3 ubiquitin protein ligase 1b	0.79/0.77	-0.27
miR-26	ENSDARG00000034643	*fhl3a*	*fd57e01, fhl3, wu:fd57e01*	four and a half LIM domains 3a	0.70/-	-0.27
miR-26	ENSDARG00000092199	*si:ch211-250c4.4*	N/A	*si:ch211-250c4.4*	0.71/0.73	-0.27
miR-26	ENSDARG00000069980	*lman1*	*fc54c09, fi36e01, wu:fc54c09, wu:fi36e01*	lectin, mannose-binding, 1	0.71/0.72	-0.27
miR-26	ENSDARG00000036584	*st8sia5*	N/A	ST8 alpha-N-acetyl-neuraminide alpha-2,8-sialyltransferase 5	0.94/0.95	-0.27
miR-26	ENSDARG00000056381	*cfap97*	*fj68c08, wu:fj68c08, zgc:85910*	cilia and flagella associated protein 97	0.81/0.77	-0.27
miR-26	ENSDARG00000068883	*tspan13a*	*zgc:91858*	tetraspanin 13a	0.82/0.81	-0.27
miR-26	ENSDARG00000060054	*epc1b*	*zgc:158364*	enhancer of polycomb homolog 1 (Drosophila) b	0.99/0.99	-0.27
miR-26	ENSDARG00000079688	*tnrc6a*	*wu:fc10d10*	trinucleotide repeat containing adaptor 6A	0.99/0.99	-0.27
miR-26	ENSDARG00000078759	*dna2*	*si:ch211-1n9.7*	DNA replication helicase/nuclease 2	0.85/0.85	-0.27
miR-26	ENSDARG00000057736	*ano10*	*ano10, fc37b04, wu:fc37b04, zgc:114140*	anoctamin 10a	0.71/0.70	-0.27
miR-26	ENSDARG00000090646	*tnk2a*	*acka, si:dkey-150o13.3*	tyrosine kinase, non-receptor, 2a	0.71/0.71	-0.27
miR-26	ENSDARG00000029724	*pqbp1*	*pqbp1l, si:dz150f13.4, zgc:92646*	polyglutamine binding protein 1	0.74/0.75	-0.26
miR-26	ENSDARG00000027590	*sept2*	*nedd5, wu:fb52g01, wu:fb99a01*	septin 2	0.76/0.75	-0.26
miR-26	ENSDARG00000021345	*prph2lb*	*prph2l, si:dkey-85n7.2*	peripherin 2-like b	0.83/0.81	-0.26
miR-26	ENSDARG00000078430	*tiam1a*	*tiam1*	TIAM Rac1 associated GEF 1a	0.74/0.73	-0.26
miR-26	ENSDARG00000004082	*cx39.9*	*cx46l, zgc:110668*	connexin 39.9	0.82/0.82	-0.26
miR-26	ENSDARG00000031929	*stard14*	*stard10, wu:fc41g06, zgc:56270, zgc:77753*	START domain containing 14	0.73/0.73	-0.26
miR-26	ENSDARG00000010347	*acer1*	*zgc:110285*	alkaline ceramidase 1	0.74/0.74	-0.26
miR-26	ENSDARG00000055752	*npas4a*	*npas4, wu:fj52d04, zgc:136764*	neuronal PAS domain protein 4a	0.74/0.74	-0.26
miR-26	ENSDARG00000045360	*flj11011l*	*zgc:136503*	hypothetical protein FLJ11011-like (H. sapiens)	0.90/0.87	-0.26
miR-26	ENSDARG00000052304	*uqcrc1*	*zgc:73404, zgc:85750*	ubiquinol-cytochrome c reductase core protein 1	0.73/0.72	-0.26
miR-26	ENSDARG00000040284	*si:dkey-79d12.5*	*BX005256.1*	*si:dkey-79d12.5*; orthologous to human *BTG3* (BTG anti-proliferation factor 3	0.82/0.80	-0.26
miR-26	ENSDARG00000045910	*tmem181*	*si:ch211-222h17.5, si:ch211-51e12.4*	transmembrane protein 181	0.87/0.87	-0.26
miR-26	ENSDARG00000055381	*bambia*	*Zfnma, bambi, cb998, etID64300.12, fc44c12, id:ibd5020, nma, wu:fc44c12, wu:fe01e03*	BMP and activin membrane-bound inhibitor (Xenopus laevis) homolog a	0.93/0.91	-0.26
miR-26	ENSDARG00000070348	*hoxc10a*	*hoxc10, z-81*	homeobox C10a	0.94/0.94	-0.26
miR-26	ENSDARG00000091539	*ptprjb.1*	*density-enhanced phosphatase-1b, dep1b, ptprjb*	protein tyrosine phosphatase receptor type Jb, tandem duplicate 1	0.77/0.78	-0.25
miR-26	ENSDARG00000075092	*si:dkey-52l18.4*	*sc:d808*	*si:dkey-52l18.4*	0.77/0.77	-0.25
miR-26	ENSDARG00000016093	*qtrt2*	*qtrtd1*	queuine tRNA-ribosyltransferase accessory subunit 2	0.73/0.75	-0.25
miR-26	ENSDARG00000062550	*rc3h1a*	*rc3h1, si:dkey-192m14.4*	ring finger and CCCH-type domains 1a	0.85/0.85	-0.25
miR-26	ENSDARG00000057699	*styx*	*si:ch211-270g19.2, zgc:110621*	serine/threonine/tyrosine interacting protein	0.74/0.76	-0.25
miR-26	ENSDARG00000040988	*tpi1b*	*DrTPI-B, cb179, wu:fb51c02*	triosephosphate isomerase 1b	0.89/0.89	-0.25
miR-26	ENSDARG00000063014	*dbpa*	*dbp1, si:dkey-109n11.1, zdbp1*	D site albumin promoter binding protein a	0.71/0.71	-0.25
miR-26	ENSDARG00000028213	*ttn.2*	*ttna, TTN2, pickwick, pik, si:busm1-258d18.1, si:dz258d18.1, ttn, ttna, wu:fi04b11, wu:fi20g08*	titin, tandem duplicate 2	0.92/0.92	-0.25
miR-26	ENSDARG00000010276	*ptgs2b*	*Cox-2b, si:dkey-97o5.6*	prostaglandin-endoperoxide synthase 2b	0.72/0.72	-0.25
miR-26	ENSDARG00000053362	*cilp*	*im:7137994*	cartilage intermediate layer protein, nucleotide pyrophosphohydrolase	0.77/0.76	-0.25
miR-26	ENSDARG00000020893	*slc25a55a*	*cb229, sb:cb229, slc25a22, wu:fa12h05, wu:fb72h03, zgc:56592*	solute carrier family 25 member 55a	0.72/0.72	-0.25
miR-26	ENSDARG00000075560	*kmt2cb*	*mll3b, si:dkey-193e13.2*	lysine (K)-specific methyltransferase 2Cb	0.77/0.76	-0.25
miR-26	ENSDARG00000073724	*nlrx1*	*NLR-A5, Nod5, Nod9*	NLR family member X1	0.80/0.78	-0.25
miR-26	ENSDARG00000088505	*fam217b*	*si:dkey-211h10.3*	family with sequence similarity 217 member B	0.71/0.71	-0.24
miR-26	ENSDARG00000088318	*ubap2a*	*ubap2, zgc:158449*	ubiquitin associated protein 2a	0.71/0.71	-0.24
miR-26	ENSDARG00000008034	*skib*	*fc17g02, skiB, wu:fc17g02, wu:fk79g04*	v-ski avian sarcoma viral oncogene homolog b	0.78/0.79	-0.24
miR-26	ENSDARG00000071548	*josd1*	*zgc:153682*	Josephin domain containing 1	0.71/0.71	-0.24
miR-26	ENSDARG00000070408	*ccnd2b*	*si:ch211-240j22.2*	cyclin D2, b	0.72/0.71	-0.23
miR-26	ENSDARG00000002192	*aspn*	*aspnl, bgl3, wu:fk08c11, zgc:109936*	asporin (LRR class 1)	0.76/0.76	-0.23

miR-26	ENSDARG00000039892	*gnpnat1*	*im:7138655, zgc:112267*	glucosamine-phosphate N-acetyltransferase 1	0.78/0.78	-0.23
miR-26	ENSDARG00000062323	*adam23a*	*wu:fj40f01, wu:fq14a08*	ADAM metallopeptidase domain 23a	0.95/0.94	-0.23
miR-26	ENSDARG00000070545	*top1l*	*wu:fc66a02, zgc:136349*	DNA topoisomerase I, like	0.87/0.86	-0.23
miR-26	ENSDARG00000025012	*tpi1a*	*DrTPI-A*	triosephosphate isomerase 1a	0.72/0.73	-0.23
miR-26	ENSDARG00000028396	*fkbp5*	*si:zc263a23.8, wu:fc31g11, wu:fl87b03, zgc:64082*	FKBP prolyl isomerase 5	0.82/0.83	-0.23
miR-26	ENSDARG00000011727	*b9d1*	*im:6905750, im:7069491, wu:fc09g07, zgc:110733*	B9 protein domain 1	0.87/0.89	-0.23
miR-26	ENSDARG00000038754	*plk3*	*cnk, wu:fi35f10*	polo-like kinase 3 (Drosophila)	0.72/0.71	-0.23
miR-26	ENSDARG00000042846	*disp2*	N/A	dispatched homolog 2 (Drosophila)	0.84/0.83	-0.23
miR-26	ENSDARG00000052975	*ubqln4*	*ataxin-1 ubiquitin-like interacting protein, ubin, wu:fb48e11, wu:fb54b10, wu:fi41e06, zgc:113928, zgc:66188*	ubiquilin 4	0.81/0.80	-0.23
miR-26	ENSDARG00000052950	*arhgap23a*	*sb:eu857*	Rho GTPase activating protein 23a	0.88/0.88	-0.22
miR-26	ENSDARG00000000142	*tmem267*	*si:busm1-79m10.4, si:dz79m10.4, zgc:101064, C10H5orf28*	transmembrane protein 267	0.77/0.77	-0.22
miR-26	ENSDARG00000003165	*nr2f6b*	*EAR2-B, fc72d04, nr2f6, wu:fc72d04, zgc:77260*	nuclear receptor subfamily 2, group F, member 6b	0.85/0.83	-0.22
miR-26	ENSDARG00000059280	*hoxd3a*	*Z-56, hoxd3, im:7144208*	homeobox D3a	0.90/0.90	-0.22
miR-26	ENSDARG00000044485	*sall4*	*cb372, cb614, ik:tdsubc_2c1, sb:cb372, sb:cb614, wu:fa01g03, xx:tdsubc_2c1*	spalt-like transcription factor 4	0.94/0.93	-0.22
miR-26	ENSDARG00000004218	*rnd1b*	*Rnd1a, rnd1l, wu:fk50c04, zgc:153089*	Rho family GTPase 1b	0.78/0.78	-0.22
miR-26	ENSDARG00000035578	*hs3st1l2*	*3-ost-5, zf3-ost-5, zgc:136835*	heparan sulfate (glucosamine) 3-O-sulfotransferase 1-like 2	0.76/0.75	-0.22
miR-26	ENSDARG00000076163	*col19a1*	*si:dkey-49l3.2, stumpy, sty, zgc:153049*	collagen, type XIX, alpha 1	0.77/0.76	-0.22
miR-26	ENSDARG00000015734	*aicda*	*Aid*	activation-induced cytidine deaminase	0.72/0.73	-0.22
miR-26	ENSDARG00000025535	*clint1a*	*clint1, enth, enthoprotin, epn4, kiaa0171, wu:fa05f05, wu:fb97f11, wu:fc12e09, zgc:103447*	clathrin interactor 1a	0.72/0.72	-0.22
miR-26	ENSDARG00000052535	*ubxn10*	*zgc:153648*	UBX domain protein 10	0.72/0.72	-0.22
miR-26	ENSDARG00000052011	*rrad*	*fi06a10, wu:fi06a10, zgc:63471*	Ras-related associated with diabetes	0.92/0.92	-0.22
miR-26	ENSDARG00000060893	*col8a2*	*si:ch211-197n10.4*	collagen, type VIII, alpha 2	0.75/0.74	-0.22
miR-26	ENSDARG00000059596	*clip2*	N/A	CAP-GLY domain containing linker protein 2	0.74/0.74	-0.22
miR-26	ENSDARG00000092692	*pimr133*	*si:dkey-197d18.1*	Pim proto-oncogene, serine/threonine kinase, related 133	0.73/0.75	-0.22
miR-26	ENSDARG00000042732	*tmem189*	*zgc:101801*	transmembrane protein 189	-/0.70	-0.22
miR-26	ENSDARG00000039064	*st3gal7*	*SIAT5-r, ST3GalV-2, st3gal5-r2, st3gal5l, wu:fb60h07, zgc:153853*	ST3 beta-galactoside alpha-2,3-sialyltransferase 7	0.84/0.84	-0.22
miR-26	ENSDARG00000003109	*rcan1a*	*zgc:100963*	regulator of calcineurin 1a	0.82/0.82	-0.22
miR-26	ENSDARG00000057497	*stk35l*	*im:7138176, zgc:101750*	serine/threonine kinase 35, like	0.78/0.78	-0.21
miR-26	ENSDARG00000036171	*rnasel3*	*Dr-RNase1, RNase ZF-3, im:6907218, zf-rnase-3*	ribonuclease like 3	0.79/0.79	-0.21
miR-26	ENSDARG00000076052	*sez6l2*	*si:dkey-282h22.6*	seizure related 6 homolog (mouse)-like 2	0.82/0.81	-0.21
miR-26	ENSDARG00000036791	*dnmt3bb.1*	*cb633, dnmt3b, dnmt3b1, dnmt4*	DNA (cytosine-5-)-methyltransferase 3 beta, duplicate b.1	0.86/0.86	-0.21
miR-26	ENSDARG00000004724	*tcea3*	*zgc:77732*	transcription elongation factor A (SII), 3	0.74/0.72	-0.21
miR-26	ENSDARG00000079783	*isg20*	*BX294110.1*	interferon stimulated exonuclease gene	0.77/0.78	-0.21
miR-26	ENSDARG00000014386	*galnt6*	*fc23d02, fc56f12, wu:fc23d02, wu:fc56f12, zgc:77836*	UDP-N-acetyl-alpha-D-galactosamine:polypeptide N-acetylgalactosaminyltransferase 6 (GalNAc-T6)	0.72/0.72	-0.21
miR-26	ENSDARG00000075757	*gig2e*	*DreE, BX510940.2*	grass carp reovirus (GCRV)-induced gene 2e	0.70/0.71	-0.21
miR-26	ENSDARG00000074365	*zgc:171901*	N/A	*zgc:171901*	0.75/0.76	-0.21
miR-26	ENSDARG00000057665	*sprn2*	*PrP-rel-1, Sho2, sprnb, wu:fj12d11, zgc:123052*	shadow of prion protein 2	0.70/-	-0.21
miR-26	ENSDARG00000073732	*myh14*	*si:ch211-120k19.2, si:ch211-120k19.3*	myosin, heavy chain 14, non-muscle	0.79/0.80	-0.21
**Total**	**513**					

*The miTG score to miR-26a and miR-26b is presented separately.
